# Identification and validation of a tear fluid-derived protein biomarker signature in patients with amyotrophic lateral sclerosis

**DOI:** 10.1186/s40478-025-02109-6

**Published:** 2025-09-02

**Authors:** Lena-Sophie Scholl, Antonia F. Demleitner, Jenny Riedel, Seren Adachi, Lisa Neuenroth, Clara Meijs, Laura Tzeplaeff, Lucas Caldi Gomes, Ana Galhoz, Isabell Cordts, Christof Lenz, Michael Menden, Paul Lingor

**Affiliations:** 1 Department of Neurology, TUM University Hospital Munich, Munich, Germany; 2 Computational Health Center, Helmholtz Munich, Neuherberg, Germany; 3 Department of Biology, Ludwig-Maximilians University, Munich, Martinsried, Germany; 4 Department of Biochemistry and Pharmacology, Bio21 Molecular Science and Biotechnology Institute, The University of Melbourne, Parkville, Victoria Australia; 5 Melbourne Bioinformatics, Faculty of Medicine, Dentistry and Health Sciences, The University of Melbourne, Parkville, Victoria Australia; 6 Department of Clinical Chemistry, University Medical Center Göttingen, Göttingen, Germany; 7 Bioanalytical Mass Spectrometry Group, Max Planck Institute for Multidisciplinary Sciences, Göttingen, Germany; 8 German Center for Neurodegenerative Diseases (DZNE), Munich, Germany; 9 Munich Cluster for Systems Neurology (SyNergy), Munich, Germany

**Keywords:** Amyotrophic lateral sclerosis, Biomarker, Tear fluid, Diagnosis, Proteomics, Neurodegeneration

## Abstract

The diagnosis of Amyotrophic Lateral Sclerosis (ALS) remains challenging, particularly in early stages, where characteristic symptoms may be subtle and nonspecific. The development of disease-specific and clinically validated biomarkers is crucial to optimize diagnosis. Here, we explored tear fluid (TF) as a promising ALS biomarker source, given its accessibility, anatomical proximity to the brainstem as an important site of neurodegeneration, and proven discriminative power in other neurodegenerative diseases. Using a discovery approach, we profiled protein abundance in TF of ALS patients (*n* = 49) and controls (*n* = 54) via data-independent acquisition mass spectrometry. Biostatistical analysis and machine learning identified differential protein abundance and pathways in ALS, leading to a protein signature. These proteins were validated by Western blot in an independent cohort (ALS *n* = 51; controls *n* = 52), and their discriminatory performance was assessed in-silico employing machine learning. 876 proteins were consistently detected in TF, with 106 differentially abundant in ALS. A six-protein signature, including CRYM, PFKL, CAPZA2, ALDH16A1, SERPINC1, and HP, exhibited discriminatory potential. We replicated significant differences of SERPINC1 and HP levels between ALS and controls across the cohorts, and their combination yielded the best in-silico performance. Overall, this investigation of TF proteomics in ALS and controls revealed dysregulated proteins and pathways, highlighting inflammation as a key disease feature, strengthening the potential of TF as a source for biomarker discovery.

**Supplementary Information:**

The online version contains supplementary material available at 10.1186/s40478-025-02109-6.

## Introduction

Amyotrophic Lateral Sclerosis (ALS) is the most common motor neuron disease, marked by progressive degeneration of both the upper (UMN) and lower motor neurons (LMN), leading to muscle weakness and atrophy [1]. ALS diagnosis primarily relies on clinical assessments of UMN and LMN dysfunction [2, 3], but its heterogeneous presentation in early stages often delays a definite diagnosis by 10–16 months after symptom onset [4], imposing a substantial burden on patients and impending prompt treatment. The prognosis for most ALS patients remains poor [5]. Early diagnosis and timely care are essential for patient quality of life, highlighting the urgent need for biomarkers to enable rapid, accurate diagnosis and to monitor disease progression and treatment response.

Previous studies have identified several biomarker candidates in cerebrospinal fluid (CSF) and blood, including neurofilaments [6], chitinases [7], TDP-43 [8], cystatin C [9], creatine kinase [10], and tau protein [11]. The most convincing data for urinary biomarkers exists for neopterin [12] and p75ECD [13]. Among these, neurofilaments and particularly neurofilament light (NfL), are the most extensively studied in neurodegenerative diseases (NDD) as they are believed to reflect axonal damage and degeneration [14]. NfL has shown promise in diagnostics [15, 16], prediction of prognosis [17], but most importantly as a surrogate marker for therapeutic response in clinical trials [18]. Recently, chitinases CHIT1, CHI3L1, and CHI3L2 have emerged as potential CSF and blood biomarkers for ALS, as their expression is associated with neuroinflammation [19], with elevated CSF chitinase levels observed in ALS patients compared to controls and ALS mimics [20].

High-throughput omics technologies, particularly proteomics, have transformed biomarker research by enabling large-scale detection of several proteins simultaneously through mass spectrometry. This advancement has facilitated the identification of differentially abundant proteins associated with ALS [21, 22]. Several of these proteins have been validated as disease biomarkers and successfully translated into clinical applications [23].

While most biomarkers have been identified in CSF and blood, both sources have limitations. Blood’s selective permeability across the blood-brain barrier restricts direct contact with the site of neurodegeneration. Although CSF contains molecules that are more relevant to central nervous system (CNS) pathology, its collection is invasive, costly, and less accepted by patients. In contrast, tear fluid (TF) is readily and rapidly accessible. Unstimulated tears can be collected using Schirmer strips, a non-invasive and cost-efficient method suitable for bedside clinical testing [24]. 

TF production is stimulated by parasympathetic fibers from the superior salivatory nucleus in the brainstem [25]. This anatomical connection, along with TF being an ultrafiltrate of blood, suggests its potential as an indicator for disease-specific alterations [26]. Elevated levels of alpha-synuclein and NfL have been found in the TF of Parkinson’s disease patients compared to controls [27, 28]. Additionally, Alzheimer’s disease-specific biomarkers, including amyloid-beta peptides and tau proteins, have been detected in TF [29].

To investigate TF as a biomarker source in ALS, we conducted a study evaluating a protein signature for ALS prediction. Here, we present the characterization of the TF proteome in a cohort of 49 ALS and 54 control subjects using data-independent mass spectrometry (DIA-MS). A protein signature was identified through biostatistical and machine learning models and subsequently validated with Western blot analysis and machine learning models in an independent cohort of 51 ALS and 52 controls.

## Methods

### Tear fluid (TF) collection

TF was collected between September 2019 and June 2022 in the in- and outpatient clinics of the Department of Neurology, TUM University Hospital, Germany. The study adheres to the Declaration of Helsinki of the World Medical Association. Permission was obtained from the Ethics Committee of the Technical University of Munich, School of Medicine (approval number: 9/15S). Unstimulated TF was collected with Schirmer strips (Madhu Instruments Pvt. Ltd., New Delhi, India) according to a standardized protocol [30]. Prior to sampling, participants were educated about the procedure, and written informed consent was acquired. For the sampling process, the upper part of the strip was inserted into the lower conjunctival sac at a one-third distance from the lateral canthus of both eyes. TF collection lasted 5 min per patient. Once finished, the Schirmer strips were removed, and the wetting length (WL) in millimeters was recorded for each eye. The strips were then separately transferred into collection tubes and kept on ice before being immediately stored at -80 °C until further use.

### Study population

**Fig. 1:**
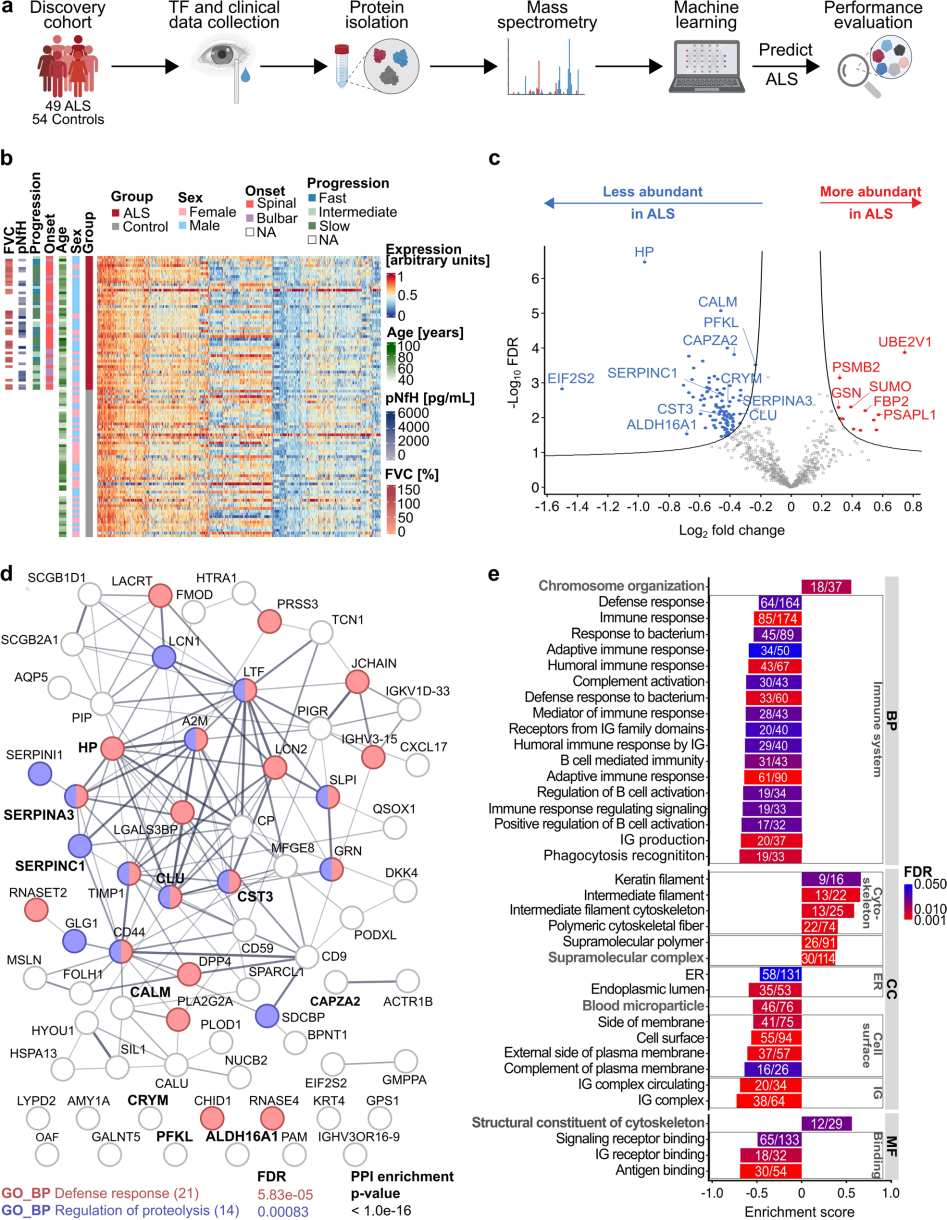
Overview of the TF proteomics results. **a** Experimental workflow for the discovery approach. TF and clinical data were collected from ALS (*n* = 49) and control subjects (*n* = 54). TF proteins were analyzed by mass spectrometry, and machine learning models were used to predict ALS/control status. Created with BioRender.com. **b** Heatmap of normalized TF protein abundance in the discovery cohort, with unsupervised hierarchical clustering of 876 consistently quantified proteins. Rows represent proteins, columns represent samples. Clinical features, including sex, age [years], onset, progression rate, CSF pNfH levels [pg/mL], and FVC [%], are annotated. **c** Volcano plot of the differential protein abundance between ALS and control subjects. x-axis represents log_2_(fold change), y-axis represents -log_10_(FDR). Significantly less abundant proteins (blue) and more abundant proteins (red) in ALS are shown. Significance was estimated by PERSEUS standard settings, including FDR < 0.1 and S0 = 0.1. **d** STRING PPI network analysis for significantly less abundant proteins. Hub proteins are associated with “defense response” (red) and “regulation of proteolysis” (purple). The number of proteins in parentheses indicates the count of proteins assigned to each pathway. PPI enrichment *p*-value (< 1.0e-16) is displayed. **e** GSEA of TF proteome in ALS and controls, showing significant GO terms for biological processes, cellular components, and molecular functions. Enrichment scores (x-axis) and annotated GO terms (y-axis) are shown. Bar colors reflect FDR, with pathway counts listed. Pathways referring to the same category were manually summarized under generic terms (bolded grey). Benjamini-Hochberg correction and an FDR cut-off of 5% were applied. **Abbreviations**: ALS, Amyotrophic Lateral Sclerosis; TF, tear fluid; FVC, forced vital capacity; CSF; cerebrospinal fluid; pNfH, phosphorylated neurofilament heavy chain; GO, gene ontology; BP, biological process; PPI, protein-protein interaction; CC, cellular component; MF, molecular function; ER, endoplasmatic reticulum; IG, immunoglobulins; HP, haptoglobin; CALM, calmodulin; PFKL, phosphofructokinase, liver type; CAPZA2, f-actin-capping protein subunit alpha-2; EIF2S2, eukaryotic translation initiation factor 2 subunit 2; SERPINC1, antithrombin-III; CRYM, mu-crystallin; SERPINA3, alpha-1-antichymotrypsin; CST3, cystatin C; ALDH16A1, aldehyde dehydrogenase family 16 member A1; CLU, clusterin; UBE2V1, ubiquitin-conjugating enzyme E2 variant 1; PSMB2, proteasome subunit beta type-2; SUMO, small-ubiquitin modfier; GSN, gelsolin; FBP2, fructose-1,6-bisphosphatase isozyme 2; PSAPL1, proactivator polypeptide-like 1

For the discovery cohort, 49 patients with probable or definite ALS, according to the revised El Escorial criteria, were selected and compared to 54 age-matched control subjects without evidence of neurodegeneration (Fig. 1a). Due to the exploratory nature of this analysis, the sample size was determined based on previously published cohort sizes as well as the availability of samples in a rare disease [26]. Since secreted tear volume is known to be positively correlated with tear protein concentration [31, 32], only TF samples with sufficient WL (≥ 5 mm) were included in the study.

An independent validation cohort, comprising 51 ALS and 52 age- and sex-matched control patients without signs of neurodegenerative disease adhering to the same inclusion criteria as those of the discovery cohort, was selected. The demographic and clinical characteristics of the validation cohort were matched to those of the discovery cohort (Table 1).

**Table 1:** Demographic and clinical data of the discovery and validation cohort

	Discovery cohort			Validation cohort			DC vs. VC
	**Controls**	**ALS**	*p*-value	**Controls**	**ALS**	*p*-value	*p*- value
**Demographics**							
Patients *n*	54	49		52	51		
Male / female *n* (% female)	22 / 32 (58.26)	35 / 14 (28.57)	0.003^a^	29 / 23 (44.23)	27 / 24 (47.06)	0.844^a^	0.020^c^
Age [years]							
Median (IQR)	60 (48, 74)	60 (53, 73)	0.302^b^	63 (57, 76)	64 (57, 75)	0.832^b^	0.094^d^
**Disease-specific parameters**							
Subgroup							1 ^a^
sALS *n* (%)	NA	47 (95.92)		NA	48 (94.12)		
fALS *n* (%)	NA	2 (4.08)		NA	3 (5.88)		
Gene mutation							0.098 ^a^
C9orf72 *n* (%)	NA	1 (2.04)		NA	6 (11.77)		
SOD1 *n* (%)	NA	1 (2.04)		NA	0 (0)		
Variants associated with ALS *n* (%)	NA	1 (2.04)		NA	2 (3.92)		
VUS *n* (%)	NA	1 (2.04)		NA	3 (5.88)		
Not determined *n* (%)	NA	36 (73.45)		NA	26 (50.98)		
Stratum of onset							1^a^
Spinal *n* (%)	NA	34 (69.39)		NA	36 (70.59)		
Bulbar *n* (%)	NA	15 (30.61)		NA	15 (29.41)		
Disease duration [years] at TF collection							0.247 ^b^
Median (IQR)	NA	20 (9, 37)		NA	17 (12, 20)		
ALSFRS-R at TF collection							0.177^e^
Median (IQR)	NA	35 (29, 41)		NA	37 (31, 42)		
Progression rate							0.135^a^
Fast *n* (%)	NA	10 (20.41)		NA	8 (15.69)		
Intermediate *n* (%)	NA	12 (24.45)		NA	22 (43.14)		
Slow *n* (%)	NA	26 (53.06)		NA	18 (35.29)		
Not determined *n* (%)	NA	1 (2.04)		NA	3 (5.88)		
CSF pNfH [pg/mL]							0.135^b^
Median (IQR)	NA	1,931 (1,175, 3,155)		NA	2,830 (1,454, 4,744)		
Not determined *n* (%)	NA	21 (42.86)		NA	13 (25.49)		
FVC [%]							0.703^e^
Median (IQR)	NA	84 (65, 93)		NA	77 (55, 98)		
Not determined *n* (%)	NA	20 (40.82)		NA	16 (31.38)		
**Ophthalmological parameters**							
Cumulative WL of both strips [mm/5 min]							
Median (IQR)	20 (9, 35)	15 (10, 30)	0.486^b^	21 (11, 30)	19 (11, 28)	0.651^b^	0.749 ^d^
Eye disease *n* (%)	11 (20.37)	5 (10.20)	0.182^a^	12 (23.08)	16 (31.37)	0.382 ^a^	0.079 ^c^
Topical eye substances *n* (%)	6 (11.11)	3 (6.12)	0.493^a^	3 (5.77)	2 (3.92)	1 ^a^	0.549 ^a^
Contact lenses *n* (%)	3 (5.56)	1 (2.04)	0.619^a^	1 (1.92)	4 (7.84)	0.205 ^a^	0.430 ^a^

**Abbreviations**: DC, discovery cohort; VC, validation cohort; IQR; interquartile range; ALS, amyotrophic lateral sclerosis; sALS, sporadic ALS; fALS, Familial ALS; VUS, variant of uncertain significance; TF, tear fluid; ALSFRS-R, ALS functional rating scale revised; CSF, cerebrospinal fluid; pNfH, phosphorylated neurofilament heavy chain; FVC, forced vital capacity in percent of normal; WL, wetting length; NA, not applicable

^a^Fisher’s exact test ^b^Mann Whitney test ^c^ Chi-square test ^d^Kruskal-Wallis test ^e^Unpaired t-test

### Demographic and clinical data analysis

The following clinical data were extracted from medical records and interviews: age, sex, eye diseases, eye medication and/or lubricants, and the use of contact lenses. For ALS patients, we additionally assessed ALS-associated genetic variants, the time point of first symptoms and diagnosis, and the revised ALS Functional Rating Scale (ALSFRS-R) score at two time points (first recorded and at TF sampling). According to initial symptoms, patients were divided into two phenotypic groups: spinal and bulbar onset. Disease duration was calculated as the difference between the self-reported appearance of symptoms and the date of TF collection. Disease severity was assessed by the ALSFRS-R sum score [33] and the forced vital capacity (FVC, expressed as a percentage of normal) at the time of TF sampling. Disease progression was determined by calculating the difference between the ALSFRS-R at symptom onset and the first recorded ALSFRS-R divided by the time (in months) between both visits (ΔFRS) [34]. ALS patients were assigned accordingly to three subgroups: fast progressors (ΔFRS > 1.11 points/month), intermediate progressors (ΔFRS 0.47–1.11 points/month), and slow progressors (ΔFRS < 0.47 points/month) [35]. CSF levels of phosphorylated neurofilament heavy chain (pNfH) were measured as part of routine diagnostic procedures when the diagnosis of ALS was suspected.

### Mass spectrometry (MS) sample preparation

Proteomic analyses were performed in a blinded manner without knowledge of the cohort affiliation of the samples. Proteins were extracted from Schirmer strips without additional washing or pre-conditioning steps to avoid selective loss of soluble proteins. Individual strips were extracted overnight using RIPA buffer augmented with a phosphatase inhibitor cocktail (Roche, Mannheim, Germany) at 37 °C, 800 rpm in a thermoshaker. Following centrifugation (17,000 x G, 4 °C, 30 min), the supernatant was collected, and protein concentration was determined by bicinchoninic acid (BCA) assay. Aliquots containing 20 µg protein were filled up with 100 mM aqueous ammonium bicarbonate pH 8.0 to a volume of 80 µL. Proteins were reduced and alkylated with tris-(2-carboxyethyl)phosphine (TCEP) (20 mM) and iodoacetamide (50 mM) in a thermoshaker (600 rpm, 37 °C, 30 min, in the dark). A modified SP3 protocol [36] was used to perform protein digestion with trypsins. 20 µg paramagnetic beads (SpeedBeads magnetic carboxylate modified, Cytiva, Marlborough, USA) were mixed with the samples in 0.2 mL tubes, and 100% ethanol (Merck, Darmstadt, Germany) was added to an ethanol concentration of 50% (volume per volume (v/v)). The samples were incubated in a thermoshaker (800 rpm, 24 °C, 5 min), then transferred to a magnetic rack, and magnetic beads captured on the magnets for 2 min. The supernatant was removed, and the bound beads were washed three times with 80% ethanol in water. Samples were allowed to air-dry, then removed from the rack and incubated with porcine trypsin (Promega, Madison, USA) in 100 mM ABC at an enzyme-to-protein ratio of 1:20 (600 rpm, 37 °C, overnight) for digestion. Following digestion, the supernatant was removed, the beads washed again with 100 mM ABC, and the combined supernatant and wash solution acidified with 10% aqueous trifluoroacetic acid (TFA) to a final TFA concentration of 0.4%. The acidified samples were then desalted on C18 tips (Pierce, Rockford, USA), and the eluates were dried in a SpeedVac and stored at -20 °C until analysis.

### MS analysis

For the generation of an MS/MS spectral library, equal aliquots of each sample were pooled and fractionated on a high-performance liquid chromatography system by basic pH reversed-phase C18 chromatography (Thermo Hypersil Gold C18 150 × 1.0 mm, gradient 5–35% aqueous acetonitrile pH 8.0, 200 µL min^− 1^). The sample was fractionated into 12 fractions using a concatenated pooling scheme, which were used to generate the library. For MS analysis, samples and reverse phase fractions were taken up in 20 µL loading buffer (0.1% aqueous formic acid, 2% acetonitrile), spiked with iRT peptides (Biognosys, Schlieren, Switzerland) [37], and separated on a nanoElute chromatography system (Bruker, Bremen, Germany). Peptides were enriched on a reversed-phase pre-column (0.3 mm ID x 5 mm PepMap C18 5Å, Thermo Fisher Scientific, Waltham, USA) and separated on an analytical RP-C18 column (0.075 mm ID x 250 mm Aurora C18, IonOpticks, Collingwood, Australia) using a 60 min (data-dependent acquisition, DDA) or 30 min (data-independent acquisition, DIA) linear gradient of 5–35% acetonitrile/0.1% formic acid (v/v) at 300 nL min^− 1^. The eluents were analyzed on a hybrid ion mobility-quadrupole-time of flight mass spectrometer (timsTOF Pro 2, Bruker, Bremen, Germany). Reversed phase fractions were then analyzed by DDA using a DDA-PASEF acquisition method [38], with a survey scan of *m/z* 100–1700 and an ion mobility enrichment of charges states 2 + to 4+. Precursors selected for MS/MS were isolated at 1.5 FWHM resolution and fragmented using nitrogen as a collision gas using default collision energy settings and up to ten repetitions per precursor depending on intensity. Four replicate injections per reversed phase fraction were analyzed. For quantitative DIA-MS, MS/MS data were acquired using 32 evenly–sized windows staggered in the ion mobility dimension to cover the 2 + to 4 + precursor population across the 400–1250 *m/z* range (DIA-PASEF) [39]. Fragments were produced using default collision energy settings at a cycle time of 1.9 s. Two replicate injections per biological sample were acquired.

### MS data processing

Data were analyzed using Spectronaut SW v14.2 (Biognosys, Schlieren, Switzerland) [40]. For generation of a spectral library, the DDA-PASEF data were searched against the UniProtKB Homo sapiens reference proteome v01/2020 using default settings at a false discovery rate (FDR) of 1%. Quantitative analysis of DIA-PASEF data was achieved by peak extraction on the precursor/fragment level using the above spectral library, combining six fragments to the precursor and up to 10 peptides to the protein for obtaining protein area values, each at estimated FDR levels of 1%.

### Preprocessing proteomics raw data

The proteomics raw data was filtered to remove proteins with over 66% missing values for at least one disease status. Log_2_-transformation was applied to the remaining values, followed by an imputation step done with the open-source software Perseus (v1.6.15.0) under default settings [41]. The data was then normalized using min-max scaling between 0 and 1.

### Proteomics differential abundance analysis

Differential abundance analysis was conducted with Perseus v1.6.15.0 using standard settings. Perseus assumes data to be missing not at random (MNAR), with missingness largely driven by a protein’s abundance in a given sample. It therefore imputes a normal distribution of values which is down-shifted by 1.8 standard deviations from the distribution of valid values [41]. In brief, after defining the experimental groups and handling the imputation of missing values, differential abundance analysis was performed using a permutation-based FDR method for multiple hypothesis testing, as described in the application manual. The FDR values were derived by comparing the proportion of significant results in the permuted data to those in the observed data. For the current analysis, proteins were deemed statistically differentially expressed with an FDR < 0.1 and S0 = 0.1 (Data S1).

### Gene set enrichment and protein-protein interaction network analyses

To investigate the pathways associated with the expressed proteins identified in TF, we performed gene set enrichment analysis (GSEA) using the “GSEA()” function from the R package clusterProfiler (v4.6.2) [42]. For the GSEA, we rank the proteins based on log-fold change, and the GSEA is conducted with a Benjamin-Hochberg correction and an FDR cut-off of 5%. To construct a tissue-specific background for the analysis, we utilize the C5 category from the ‘msigdbr’ (v7.5.1) package [43] and filter for proteins that we detected in our samples. Furthermore, following the recommendations of the GSEA User Guide, which advises that gene sets with as few as 2–3 genes can yield statistically significant results [44], we applied the more stringent threshold and only included pathways with a minimum gene set size of three. This approach ensured the inclusion of small but biologically meaningful gene sets while maintaining robustness in the enrichment analysis (Data S2).

Protein-protein interaction (PPI) network analysis with differentially abundant proteins was performed and visualized using the STRING database v12.0 with default settings [45]. Functionally enriched pathways for biological processes (BP) from gene ontology (GO) were highlighted and annotated to corresponding proteins.

### Biomarker identification leveraging machine learning

Unsupervised clustering analysis using uniform manifold approximation and projection (UMAP) and heatmap (R packages ComplexHeatmap v2.20, UMAP v0.2.10.0, R v4.2.3), was performed to stratify samples by disease status, sex, and age. The ALS cohort was further organized by clinical features such as stratum of onset, disease progression rate, FVC, and pNfH levels. Machine learning models, including lasso regression, support vector machine (SVM), and random forest, were applied for biomarker identification, using the caret package (v6.0-94) [46] with 80% training, 20% testing. A nested 10-fold cross-validation to estimate models’ hyperparameters as well as a 500 times bootstrapping procedure for performance assessment were employed to prevent overfitting. While group sizes were relatively balanced, stratified sampling was employed to account for the limited sample sizes and to ensure proportional representation during model training and evaluation. The models were selected based on their complementary strengths in biomarker discovery: Lasso regression offers embedded feature selection and interpretability; support vector machines (SVM) are well suited for high-dimensional classification tasks with limited sample size; and random forests, as ensemble models, are robust to overfitting and capable of capturing non-linear relationships. For linear regression, a binomial distribution with a lambda range of 0 to 100 (step size 0.1) was used. The Lasso regression model was run with nested 10-fold cross-validation. The optimal lambda value was selected through hyperparameter tuning within each inner cross-validation fold, and the corresponding model weights of the predictor variables were obtained. SVM analysis (e1071 package, v1.7-13) [47] employed both radial and linear kernels. For random forest, the tune length was set to 15, the tune grid ranged from 1 to 5, and 500 trees were used. Receiver operating characteristic (ROC) curves assessed the performance (Table S1).

To identify the promising biomarker proteins for further validation, we firstly created a tier list of proteins selected by the linear regression model during bootstrapping. Proteins selected in at least 20% of runs and with an SD lower than the mean were considered targets. Only significantly differentially abundant proteins were retained as candidates (Data S3). Next, linear regression models were built on these candidate proteins using their average feature importance from the initial analysis. The optimal protein signature was selected based on AUROC performance (Supplementary Methods).

### Protein isolation for western blotting

The upper part of the Schirmer strip, which was placed into the lower conjunctival sac, was discarded to prevent contamination with ocular mucosa proteins. The remaining Schirmer strip was cut into small fragments and transferred into 0.5 mL tubes. Subsequently, RIPA lysis buffer containing phosphatase and protease inhibitors (both Roche, Mannheim, Germany) was added, adapted to the WL of both the Schirmer strips (< 40 mm WL: 60 µL RIPA, 40–60 mm WL: 70 µL RIPA, > 60 mm WL: 80µL RIPA). The samples were then incubated for 60 min at 4 °C. The tube containing the incubated fragments was then placed into a bigger 1.5 mL tube, and the base of the smaller tube was perforated. Following centrifugation at 16,000 x G for 30 min at 4 °C, the aqueous proteinaceous phase was carefully transferred to a new tube. Finally, total protein concentration was measured using a BCA assay according to the manufacturer’s instructions.

### Western blot

Since there is no well-established housekeeping protein for TF, a pooled TF standard comprising samples from patients without ALS served for normalization. 30 µg of TF standard and samples were prepared with 100 mM Dithiothreitol (DTT) reducing agent and diluted in Novex Tris-Gylcine SDS sample buffer (both Thermo Fisher Scientific, Waltham, USA). Samples were loaded in triplicates in Novex 10–20% Tris-Glycine precast gels (Thermo Fisher Scientific, Waltham, USA) and wet-transferred to PVDF (Polyvinylidene fluoride) membranes using the mini blot module (Thermo Fisher Scientific, Waltham, USA). To detect all six proteins, the three membranes, each harboring two proteins of interest, were sectioned once at specific molecular weight markers as determined by the prestained protein ladder (Figure S1a-c). Membranes were blocked in 5% skimmed milk diluted in PBS-Tween 20 0.05% for 60 min (for CRYM, CAPZA2, SERPINC1, and HP) or 5% bovine serum albumin (BSA) for 180 min in PBS-Tween 20 0.05% (for PFKL and ALDH16A1). Subsequently, membranes were incubated in blocking buffer overnight at 4 °C with the following primary antibodies: CRYM mouse monoclonal antibody (1:100, Santa Cruz Biotechnology Cat# sc-376687, RRID: AB_11150103); SERPINC1 rabbit monoclonal antibody (1:10,000, Abcam Cat# ab126598, RRID: AB_11128908); CAPZA2 rabbit polyclonal antibody (1:500, Proteintech Cat# 15948-1-AP, RRID: AB_2070148); PFKM and PFKL rabbit monoclonal antibody (1:750, Abcam Cat# ab181064, RRID: AB_3675949); ALDH16A1 rabbit monoclonal antibody (1:1,000, Abcam Cat# ab137082, RRID: AB_2861363); HP rabbit monoclonal antibody (1:200, Abcam Cat# ab131236, RRID: AB_11157376). After washing, Horseradish peroxidase (HRP) horse anti-mouse IgG H&L secondary antibody (1:10,000, Vector Laboratories Cat# PI-2000-1, RRID: AB_2336177) was used for anti-CRYM, and HRP goat anti-rabbit IgG H&L secondary antibody (1:10,000, Vector Laboratories Cat# PI-1000-1, RRID: AB_2916034) for all the other proteins. Membranes were washed, incubated with Super Signal West Pico PLUS Chemiluminescent Substrate (Thermo Fisher Scientific, Waltham, USA), and developed in a Fusion SL chemiluminescence imaging system (Vilber Lourmat, Collégien, France) for protein detection (full-length as well as complete Western blots of the six proteins are provided in Figure S1d-i and Figure S2). Band intensities of the specific proteins were quantified automatically with ImageJ/Fiji2 (Version 2.1.0/1.53c) without knowledge of the cohort affiliation [48]. Signal intensities were normalized to the pooled standard to account for inter-membrane variability, and subsequently to the total protein amount of each sample to normalize to the different amounts of protein entailed by the different collection volumes.

### Statistical analysis

GraphPad Prism (Version 10) and RStudio (version 2024.12.0.467 [49]; R version 4.4.2 (2024-10-31), R Core Team [50]) were used for statistical analysis. The significance level for all analyses was set to α = 0.05 unless stated otherwise. Regarding the clinical and demographic cohort information, quantitative data are shown as median and interquartile range (IQR: Q1, Q3). Categorical data are presented as absolute and relative frequencies. Quantitative data were tested for normality with the D’Agostino-Pearson, and Shapiro-Wilk tests. The Mann-Whitney test was used for non-parametric data. Unpaired Student’s t-test was used for parametric data. Fisher’s exact test was used for categorical data, such as sex, gene mutations, stratum of onset, progression rate, or ophthalmological characteristics within the cohorts. For inter-cohort comparisons between the discovery and validation cohorts, Kruskal-Wallis testing was performed to compare multiple quantitative data. The Chi-Square test was used for the comparison of qualitative data. Concerning the Western blot validation data, log_10_-transformed protein abundance data are represented as median and IQR (Q1, Q3). Missing values were imputed using mindet imputation (CRYM ALS *n* = 5, control *n* = 4, CAPZA2 ALS *n* = 2, control *n* = 1, ALDH16A1 ALS *n* = 2, control *n* = 1, PFKL ALS *n* = 2, control *n* = 3, SERPINC1 ALS *n* = 2, control *n* = 0, HP no missing). The differences in protein abundance levels between ALS and control subjects were calculated using the Student’s t-test after testing for normality and Bonferroni for multiple testing correction.

### In-silico validation of the western blot results

Based on our Western blot validation, we employed machine learning to evaluate the performance of individual proteins as a signature for differentiating ALS from controls. We trained linear regression (lasso), SVM with a linear kernel, and random forest using the same approach as previously described for biomarker identification. The normalized Western blot abundance data with mindet imputation served as input. Embedded feature selection within the lasso model was employed to systematically identify the most important proteins for prediction. AUROCs were quantified to assess protein performances across different applied models.

## Results

### Cohorts and data description

This study included a discovery and validation cohort (Table 1). In the discovery cohort, we characterized the TF-proteome of 49 subjects with ALS and 54 control subjects without evidence of neurodegeneration (Fig. 1a), matched for age and Schirmer strip wetting length (WL). The ALS group comprised 34 spinal- and 15 bulbar-onset cases with varying progression rates (10 fast, 12 intermediate, 26 slow, 1 not determined). Median disease duration was 20 months (interquartile range (IQR): 9, 37 months), and median ALSFRS-R at TF collection was 35 months (IQR: 29, 41 months).

The independent validation cohort consisted of TF samples from 51 ALS patients and 52 control subjects, with no significant differences in age and WL between groups. To address the male overrepresentation in the ALS group of the discovery cohort (*p* = 0.003, Fisher’s exact test), the validation cohort was balanced for sex. ALS patients included 36 spinal- and 15 bulbar-onset cases with varying progression rates (7 fast, 22 intermediate, 18 slow, 3 not determined). Median disease duration was 17 months (IQR: 12, 20 months), and median ALSFRS-R at TF collection was 37 months (IQR: 31, 42 months). Apart from sex distribution, no significant differences in the demographic and clinical features were observed between cohorts.

### Individual proteomic profiles of ALS patients and control subjects

DIA-MS identified 2,205 proteins across the TF of all ALS and control subjects (FDR ≤ 0.01). Of these, 876 proteins were consistently detected and quantified in at least 2/3 (≥ 66%) of the samples per group and were used for further analysis. Unsupervised hierarchical clustering and uniform manifold approximation and projection (UMAP) did not reveal a distinct separation between ALS and controls (Figure S3; S4) or stratification by age or sex (Figure S4). In ALS patients, clustering showed no associations with demographics or clinical variables, including age, sex, genetic status, stratum of onset, disease progression rate, FVC, or pNfH levels (Fig. 1b; Figure S5). This suggests that molecular diversity in this cohort of ALS patients may be independent of demographic and clinical parameters.

### Functional enrichment analysis highlights immunological pathways in ALS

Differential abundance analysis using Perseus identified 106 significantly dysregulated proteins in ALS: 94 proteins (89%) were less abundant, whereas 12 proteins (11%) were more abundant in ALS compared to controls (Fig. 1c). Notably, we identified proteins shown to be differentially regulated in ALS before [9, 21, 51–54], e.g., cystatin C (CST3: -log_10_ FDR = 2.11, log_2_ fold change (FC) = -0.41, FC = -1.33), calmodulin (CALM: -log_10_ FDR = 5.08, log_2_ FC = -0.46, FC = -1.38), alpha-1-antichymotrypsin (SERPINA3: -log_10_ FDR = 2.50, log_2_ FC = -0.34, FC = -1.27) and clusterin (CLU: -log_10_ FDR = 2.10, log_2_ FC = -0.38, FC = 1.30) were significantly lower abundant, while gelsolin isoform 2 (GSN: -log_10_ FDR = 2.30, log_2_ FC = 0.31, FC = 1.24) was more abundant in ALS TF compared to controls (Data S1).

STRING analysis was used to investigate interactions among differentially abundant proteins in ALS. Protein-protein interaction (PPI) network analysis of proteins with reduced abundance in ALS revealed multiple hub proteins, including haptoglobin (HP), CLU, SERPINA3, CST3, and antithrombin-III (SERPINC1). The number of interactions was significantly higher than expected (*p* < 1.0e-16), indicating strong biological relation (Fig. 1d). Subnetworks enrichment analysis revealed the GO biological processes (BP) “regulation of proteolysis” (FDR = 0.00083) and “defense response” (FDR = 5.83e-05; Fig. 1d). No significant PPI were found for proteins with increased abundance in ALS (*p* = 0.295; Figure S6).

Gene set enrichment analysis (GSEA) was performed to systematically investigate biological processes (BP), cellular components (CC), and molecular functions (MF) that differed between ALS and control TF protein compositions. GO terms were annotated for all expressed proteins. Of the 37 pathways analysed, 29 (78%) were significantly downregulated in ALS, primarily related to immune system functions (Fig. 1e; Data S2). Multiple pathways related to the same generic category were manually summarized (Fig. 1e, Data S2).

Among the BP significantly altered in ALS, 17 out of 18 pathways linked to the “immune system” were downregulated. The most significantly downregulated pathways included immune response (Normalized enrichment score (NES) = -1.95, p_adj_ = 0.0005) and adaptive immune response (NES = -1.67, p_adj_= 3.77e-06). Notably, four pathways (23.5%) were associated with B cell-mediated processes, and five pathways (29.4%) were related to humoral immune response.

Focusing on CC, we observed a significant enrichment of the “cytoskeleton” category in ALS, particularly pathways associated with intermediate filaments (intermediate filaments NES = 2.25, p_adj_ = 0.0001; intermediate filament cytoskeleton NES = 2.09, p_adj_ = 0.0002) and keratin filaments (NES = 2.08, p_adj_ = 0.0001).

Regarding MF, pathways related to “binding” were downregulated, highlighting a dysregulation of immune processes such as antigen binding (NES = -2, p_adj_ = 8.34e-06) and immunoglobulin receptor binding (NES = -1.86, p_adj_ = 0.012) pathways. Conversely, the “structural constituent of cytoskeleton” category exhibited significant enrichment.

The most abundant protein family in ALS was immunoglobulins, which were represented in four of the ten generic categories (40%): “immune system”, “cell surface”, “immunoglobulins”, and “binding”. Indeed, the majority of proteins identified in the GSEA belonged to the immunoglobulin family. Among the other proteins associated with reduced immune system pathways, we identified several protein superfamilies, including complement factors (C3, C4BPA, C9 and CD55), serine protease inhibitors (SERPING1 and SERPINA3), fibrinogens (FGA and FGB), and apolipoproteins (APOB and APOE), but also proteins like haptoglobin (HP) and angiogenin (ANG).

### Machine learning distinguishes ALS from controls based on the TF proteome

**Fig. 2:**
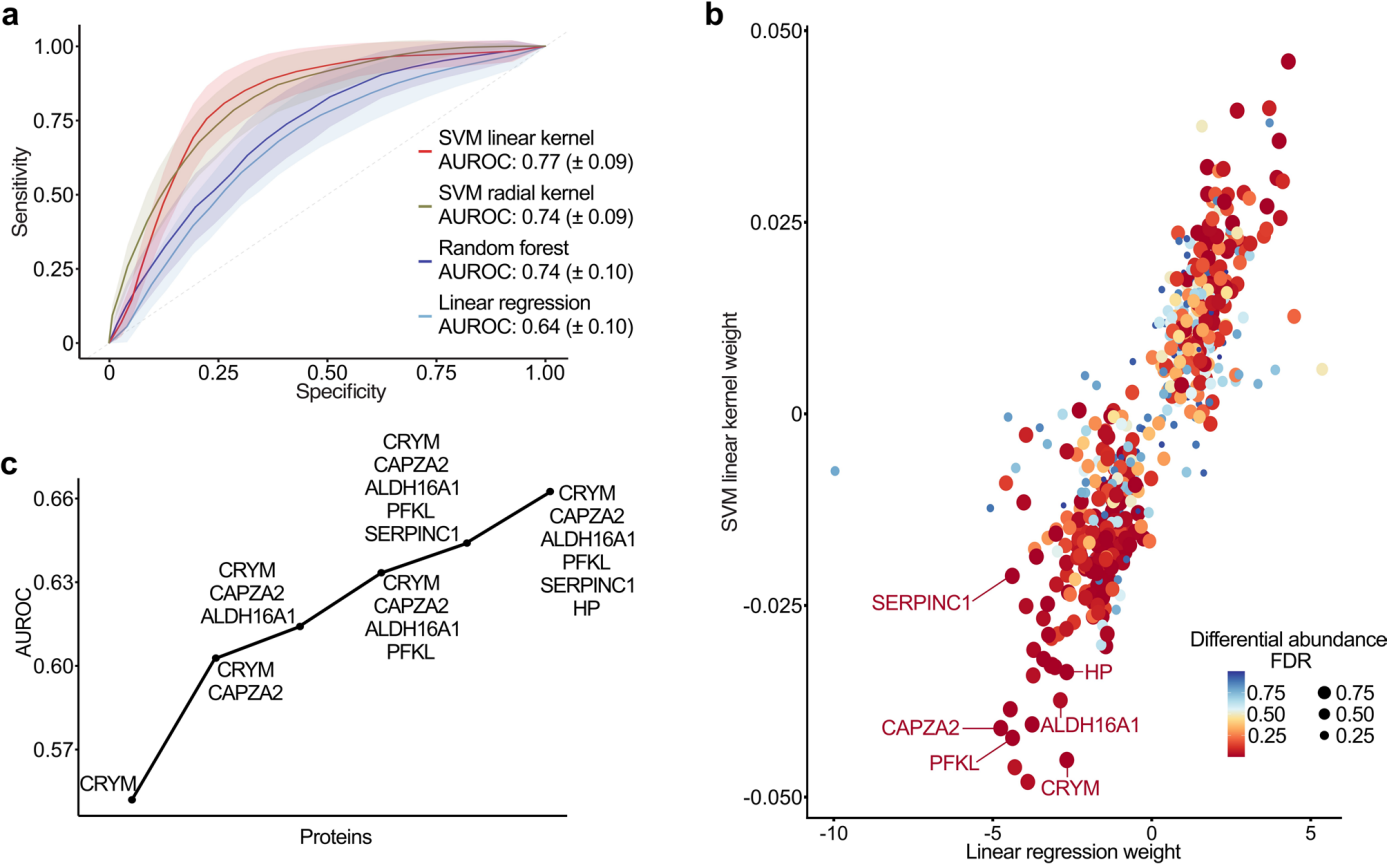
Leveraging machine learning to identify a six-protein signature for optimal discrimination between ALS and controls. **a** ROC curves for four machine learning algorithms (SVM with linear and radial kernels, random forest, and linear regression) to differentiate ALS from controls. The x-axis represents specificity, the y-axis sensitivity, and performance is quantified by AUROC, with mean ± SD and the 95% confidence interval shaded. **b** Protein importance for ALS/control classification, as determined by SVM linear kernel and linear regression, alongside differential abundance. The x- and y-axes show averaged protein weights from 500 bootstrap runs of the linear regression and SVM models, respectively. Point color and size correspond to the FDR of the differential abundance analysis. **c** AUROC curve for the six-protein signature optimized for ALS/control discrimination. The x-axis represents proteins selected by the linear regression model, and the y-axis shows the corresponding AUROC values. **Abbreviations**: SVM, support vector machine; AUROC, area under the receiver operating characteristics curve; FDR, false discovery rate; CRYM, mu-crystallin; CAPZA2, f-actin-capping protein subunit alpha-2; ALDH16A1, aldehyde dehydrogenase family 16 member A1; PFKL, phosphofructokinase, liver type; SERPINC1, antithrombin-III; HP, haptoglobin

Using the DIA-MS data, we aimed to identify a protein signature distinguishing ALS from controls. Four supervised machine learning algorithms were employed: linear regression, random forest, and support vector machine (SVM) with linear kernel or radial kernel, trained on the abundance data of the previously identified 876 TF proteins. Receiver operating characteristic (ROC) curves were generated in order to evaluate the performance via area under the ROC (AUROC). The SVM with linear kernel performed best, with an AUROC of 0.77 (± 0.09), sensitivity of 0.81 (± 0.11) and specificity of 0.74 (± 0.10), outperforming the other models (Fig. 2a, Table S1).

Despite differences in AUROC performance, the averaged feature importance assigned by the SVM (the highest-performing model) and linear regression (the lowest-performing model) for disease classification were strongly correlated, suggesting that both relied on a shared set of discriminative proteins to differentiate disease status (Fig. 2b). Although the SVM achieved slightly higher performance, the difference was negligible. Based on this concordance and the interpretability and embedded feature selection of the Lasso regression model, we prioritized its results for the identification of a protein signature.

### A six-protein signature in TF differentiates ALS from controls

To identify a more concise subset of biomarkers with high discriminatory diagnostic power, we extracted the averaged protein weights of the previously mentioned linear regression model, attributed to each protein across all 500 bootstrap runs. The models used a total of 83 proteins for the classification of ALS or control subjects. Among these, 24 were significantly differential abundant (FDR < 0.1), making them suitable for further validation as potential biomarkers (Data S3).

To identify a biomarker signature that could be suitable for clinical routine, we evaluated the performance of several linear regression models based on combinations of the previously selected 24 proteins. The optimal performance with an AUROC of 66.2% was achieved with the following six proteins: mu-crystallin (CRYM), phosphofructokinase, liver type (PFKL), f-actin-capping protein subunit alpha-2 (CAPZA2), aldehyde dehydrogenase family 16 member A1 (ALDH16A1), SERPINC1, and HP (Fig. 2c, detailed identification of the six-protein signature is provided in Supplementary Methods). All these six proteins were found to be significantly less abundant in the TF of ALS patients (Fig. 1c) and exhibited high absolute weights distinguishing ALS from controls (Fig. 2b).

### Validation of HP and SERPINC1 in an independent cohort

**Fig. 3:**
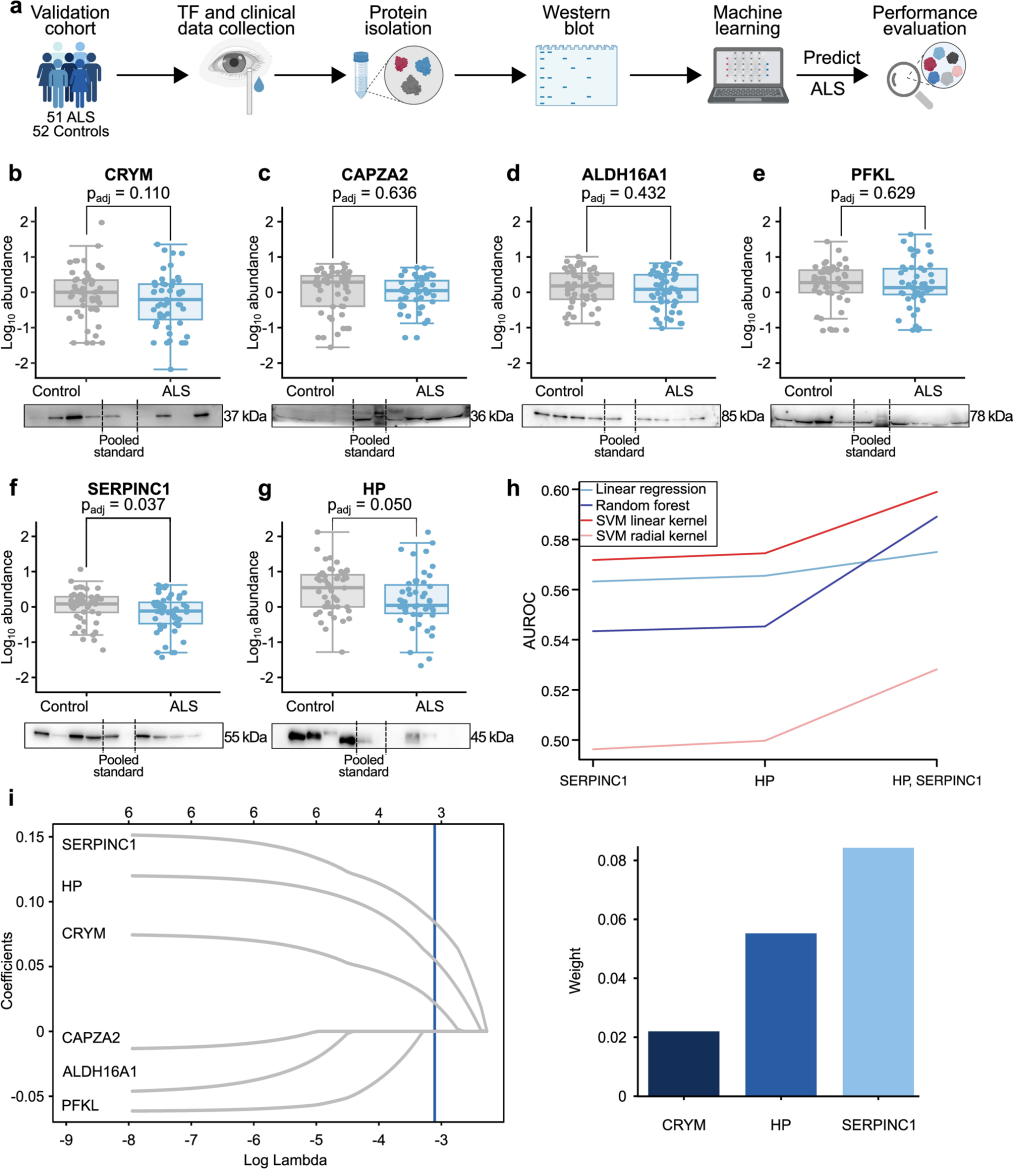
Validation of the identified six-protein signature in TF. **a** Experimental workflow for validating the identified TF protein signature. TF and clinical data were collected from an independent cohort of ALS (*n* = 51) and control (*n* = 52) subjects. TF proteins were analyzed by Western blot, followed by machine learning models to predict ALS/control status and evaluate the diagnostic potential of the proteins. Created with BioRender.com. **b-g** Western blot quantification of CRYM, CAPZA2, ALDH16A1, PFKL, SERPINC1, and HP. Boxplots show log_10_-transformed and normalized protein abundance, standardized to a pooled TF sample and the total protein amount of the individual sample. The x-axis represents ALS (*n* = 51) and control (*n* = 52) groups, and the y-axis displays log_10_-transformed normalized abundance. Statistical significance is estimated with Student’s t-test and Bonferroni correction. Blots of controls (*n* = 4), pooled TF standard (*n* = 1), and ALS (*n* = 4) are displayed. Full blots in Figure S1d-i and Figure S2. **h** Performance of four machine learning algorithms — SVM (linear + radial kernel), random forest, and linear regression — utilizing Western blot data (SERPINC1, HP and HP and SERPINC1 together) to discriminate ALS from controls. The x-axis represents proteins selected from linear regression models based on the highest weights, while the y-axis shows the corresponding AUROC values, reflecting discriminatory performance. **i** Parameterization of L1 regularization for lasso in one model from the 10-fold cross-validation. The x-axis displays log-transformed lambda values, and the y-axis shows model coefficients. The best-performing lambda is indicated (blue line). **j** The selected lasso model from **i** identifies SERPINC1, HP, and CRYM as key proteins distinguishing ALS from controls. The bar chart shows the weights (y-axis) assigned to each protein (x-axis) in the chosen lasso model. **Abbreviations**: ALS, Amyotrophic Lateral Sclerosis; TF, tear fluid; CRYM, mu-crystallin; CAPZA2, f-actin-capping protein subunit alpha-2; ALDH16A1, aldehyde dehydrogenase family 16 member A1; PFKL, phosphofructokinase, liver type; SERPINC1, antithrombin-III; HP, haptoglobin; p_adj_, adjusted *p*-value; SVM, support vector machine; AUROC, area under the receiver operating characteristics curve

To validate the hypothesis that the protein signature comprising CRYM, PFKL, CAPZA, ALDH16A1, SERPINC1, and HP can distinguish ALS from control subjects, we quantified these six proteins using Western blots in an independent validation cohort (Fig. 3a). All six proteins were detectable in TF of ALS and controls (Fig. 3b-g). Log_10_-transformed normalized abundance of SERPINC1 (ALS: -0.12, IQR: -0.48, 0.13; controls: 0.08, IQR: -0.18, 0.29; p_adj_ = 0.037) and HP (ALS: 0.04, IQR: -0.21, 0.66; controls: 0.54, IQR: 0, 0.91; p_adj_ = 0.050) was significantly lower in ALS compared to controls. A directional difference was observed for CRYM, with lower values in ALS patients (ALS: -0.20, IQR: -0.86, 0.24; controls: 0 IQR: -0.45, 0.35; p_adj_ = 0.110). Although ALDH16A1 (ALS: 0.08, IQR: -0.27, 0.50; controls: 0.18, IQR: -0.20, 0.56; p_adj_ = 0.432), CAPZA2 (ALS: 0.05, IQR: -0.26, 0.38; controls: 0.29, IQR: -0.40, 0.47; p_adj_ = 0.636), and PFKL (ALS: 0.13, IQR: -0.08, 0.68; controls: 0.27, IQR: -0.01, 0.64; p_adj_ = 0.629) showed numerically lower abundance in ALS, these differences were not statistically significant.

To confirm the performance of SERPINC1 and HP, we trained four models, SVM linear and radial kernel, lasso regression, and random forest, with data including SERPINC1 alone, HP alone, and HP and SERPINC1 combined (Fig. 3h). The three models all showed improved AUROC scores (Table S2) when data included both SERPINC1 and HP (SVM linear kernel: 0.60, SVM radial kernel: 0.53, lasso regression: 0.58, random forest: 0.59) than SERPINC alone (SVM linear kernel: 0.57, SVM radial kernel: 0.50, lasso regression: 0.56, random forest: 0.54) or HP alone (SVM linear kernel: 0.57, SVM radial kernel: 0.50, lasso regression: 0.57, random forest: 0.55).

To evaluate the contribution of all six proteins in an embedded feature selection process, we utilised the lasso regression model with L1 regularization. This approach allows for automatic feature selection while evaluating the importance of each protein in distinguishing ALS patients from controls. SERPINC1 emerged as the most consistently selected protein, followed by HP (Fig. 3i, j; Figure S7). Additionally, CRYM was identified in half of the 10 nested 10-fold cross-validation iterations, while PFKL was selected in three iterations, demonstrating the efficacy of lasso regression in identifying key biomarkers.

## Discussion

Improved biomarkers are urgently needed to enhance the accuracy of ALS diagnosis and monitor disease progression. In this context, we investigated the potential of TF, a readily accessible biofluid, as a source of protein biomarkers for ALS. In a discovery approach, we characterized individual TF proteomes in ALS patients and control subjects without indication of neurodegenerative disease. We identified multiple proteins and pathways with differential regulation between the groups. Six proteins were subsequently evaluated in an independent validation cohort to assess their potential as individual and combined biomarkers for ALS.

Importantly, we did not detect any differences in tear fluid production, in the presence of ocular diseases or medication between the different groups of our cohort, suggesting these did not significantly influence the results in our study. It is important to acknowledge, however, that ocular conditions (e.g. dry eye disease) or environmental factors (e.g. humidity) influence tear fluid production [55–57] and contents such as proteins or miRNA [58, 59].

In our discovery cohort, the higher number of males in the ALS group (Table 1) reflects the higher prevalence of ALS in males [60]. We did not find other statistical differences regarding demographic or clinical features between the groups. Phenotypic heterogeneity poses a considerable challenge in understanding ALS [61]. Clinical phenotypes (e.g., site of onset, propagation pattern, progression rate, or cognitive impairment), but also sex, age, and genetic status can have an impact on molecular composition in postmortem brain tissue or biofluids such as blood and CSF [62–64]. In our study, clustering based on the whole proteome did not allow for separation between the ALS and control cohorts. No molecular subgroup within the entire cohort or within ALS patients only could be attributed to any demographic or clinical parameters.

For sample preparation of our mass spectrometry dataset, we employed the single-pot solid-phase-enhanced sample preparation (SP3) approach. This method involves protein capture on paramagnetic beads, subsequent washing steps, and finally an on-bead tryptic digestion. The SP3 approach was selected as it combines efficient removal of a wide range of buffers and contaminants, is suitable to enrich low microgram amounts of proteins, and is readily automated and parallelized using magnetic bead handlers for reproducible analysis of medium-to-large sample cohorts [36]. Proteome profiling of digested samples was achieved using data-independent acquisition mass spectrometry (DIA-MS) on a hybrid ion mobility/quadrupole/time-of-flight mass spectrometer. DIA-MS is a widely used approach for the quantitative analysis of proteomes in body fluids, offering high sensitivity with consistent quantitation of peptides in complex matrices [39]. To improve protein detection, we generated and applied a hybrid spectral library containing both DDA (data-dependent acquisition) and DIA mass spectrometry of a pooled and fractionated reference sample [38, 39].

In our protein dataset, we identified a total of 2205 and 876 proteins before and after consistency filtering, respectively, which aligns with the reported range of other TF proteomics studies [65–68]. This relatively high degree of missingness can be readily explained by variations during sampling procedures, such as different Schirmer strip running lengths. Additionally, both inter-individual and diurnal variations in tear fluid composition may contribute, as total protein concentrations have been reported to range between 1 and 11 mg/ml [32, 69]. Nevertheless, approximately 1,000 proteins were consistently detected in at least 60% of the samples, with the majority exhibiting low variability. This included all proteins of our proposed biomarker signature, supporting their suitability for downstream analyses (Figure S8). In accordance to Dor et al. [68], we detected lactotransferrin (LTF), lipocalin-1 (LCN1), serum albumin (ALB), lysozyme C (LYZ), and immunoglobulin heavy constant alpha (IGHA) as the five most abundant TF proteins in our control subjects, as well as in the ALS patients (Figure S9, Data S4). Immunoglobulins, serpins, and 14-3-3 domain proteins were also present as known principal tear protein superfamilies [68].

Differential abundance analysis revealed significant differences in 106 proteins between ALS and control subjects, with 89% of them being less abundant in ALS patients. Notably, several of these significantly deregulated proteins (CST3, CLU, GSN, CALM, and SERPINA3) have been previously studied as potential ALS markers in post-mortem spinal cord [70] or biofluids, such as CSF or plasma [9, 21, 51–54]. The downregulation of immunoglobulins and immune system processes in the TF of ALS patients is one of the key findings of this analysis. Similarly, in another NDD, dysregulation of networks of proteins related to immune dysfunction was also observed in the TF of Parkinson’s disease patients [31, 71]. Dysregulation of the immune system has been recognized as a potential driver of disease mechanisms in ALS [72]. Neuroinflammatory proteins, for example CHIT1, serve as non-disease-specific markers for diagnosis and prognosis [7]. Interestingly, we could also detect chitinase family members (CHI3L1, CHI3L2) in the TF of ALS and control subjects. However, due to limited consistency in all samples (33% missing), CHI3L1 was removed during the filtering step. A suppression of immune system processes in ALS patients compared to controls has already been demonstrated in postmortem brain tissue [62]. In contrast, other studies have shown upregulation of proteins associated with immune response in ALS [73, 74]. Interestingly, immune response pathways seem to contribute to a molecular clustering of ALS patients [62, 64, 75]. Whether the inflammatory changes observed in TF primarily reflect systemic immune alterations or local ocular processes is challenging to determine. However, it is likely that both contribute to the observed protein profiles. Importantly, the prevalence of (inflammatory) eye disease did not differ between groups.

We then took advantage of machine learning models to identify a protein signature in the TF of ALS patients. In a similar approach, Bereman et al. identified proteomic biomarkers for ALS classification in CSF and plasma [54]. In our study, the best classification performance was achieved using an SVM with a linear kernel, which yielded an AUROC of 77% based on all 876 proteins. This model effectively differentiated ALS patients from controls, achieving high sensitivity (81%) and specificity (74%). The strong performance of the linear SVM model is consistent with its suitability for high-dimensional datasets (876 proteins) with limited sample sizes (approximately 100 patients), as commonly encountered in proteomics studies. To facilitate the translation of a diagnostic method into clinical routine, the signature combinations were optimized to a minimal set of six proteins, however achieving the maximal diagnostic performance. We identified a signature of six proteins with an AUROC of 66%, comprising CRYM, PFKL, CAPZA2, ALDH16A1, SERPINC1, and HP. In the discovery cohort, all of them were significantly less abundant in the TF of ALS patients compared to controls. The significantly lower levels of SERPINC1 and HP in ALS patients were validated with Western blot in an independent cohort. While the other proteins failed to reach significance, the direction of regulation was consistent with the data from the discovery cohort as well. Machine learning analysis of the Western blot validation findings resulted in SERPINC1 and HP jointly as the most reliable discriminators, having the highest impact in classifying ALS versus controls, yielding an AUROC of 60%.

SERPINC1 belongs to the serine protease inhibitor (serpin) family and is a crucial inactivator of several procoagulant targets in the blood coagulation cascade. Additionally, it has anti-inflammatory effects that can occur through both coagulation-dependent and coagulation-independent mechanisms [76]. In ALS patients’ blood compared to controls, significantly lower serum levels of SERPINC1 have been observed [77]. Another study identified it among the top five proteins reduced in the plasma of ALS patients [54]. In the CSF of ALS patients, SERPINC1 was identified as having a strong impact on the differentiation between fast and slow disease progression, with high SERPINC1 expression levels being associated with faster progression [64].

As a plasma protein, HP binds free hemoglobin (HB) and therefore prevents HB-induced oxidation. As an acute-phase protein, HP also regulates immune responses and exhibits antibacterial activity by limiting iron and heme utilization through microorganisms [78]. The implications of HP in ALS remain largely unexplored. Elevated HP levels have been observed in the serum of ALS patients compared to controls [79, 80]. Interestingly, in line with our findings in TF, HP levels in the CSF of ALS patients were found to be decreased compared to controls [22]. Differential abundance of HP has also been demonstrated in biofluids across multiple NDD, including Alzheimer’s disease, Huntington’s disease, Parkinson’s disease, and frontotemporal dementia [81–85]. Oxidative stress and neuroinflammation are recognized as pivotal hallmarks of NDD pathogenesis. Our findings underscore that the role of HP needs further investigation in ALS, given its involvement in neuroinflammation and various NDD.

CRYM is involved in thyroid hormone metabolism and serves as ketimine reductase in lysine degradation in the brain in the pipecolate pathway [86]. Its protein expression is indeed predominantly observed in the brain, with the highest levels detected in the basal ganglia and cerebral cortex [87, 88], followed by the kidney, prostate, and skin [89]. CRYM has been shown to be specifically expressed in some mice corticospinal motor neurons (CMNS) and subcerebral neurons of layer V in comparison to callosal and corticotectal projection neurons. Its expression, observed during intermediate stages of CMNS development in mice, suggests a potential role in the early specification and differentiation of these neuronal subtypes [90]. Interestingly, the screening of sporadic and familial ALS patients with a multi-gene panel targeting those genes associated with CMNS development identified a missense variant in a highly conserved region of the CRYM gene in cases of sALS [91]. Hommyo et al. used immunohistochemistry on post-mortem brain and spinal cord tissue samples of ALS patients, finding a progressive cranial-to-caudal decline in CRYM expression. They noted a transition from atrophic large pyramidal cells with both CRYM-positive and -negative neurons to a complete loss of CRYM expression in the distal pyramidal tracts. The authors postulated a “dying back” hypothesis with neurodegeneration starting distally, considering CRYM as a surrogate reflecting axonal degeneration in the corticospinal tract [92].

While the other proteins of the signature did not reach significance in our validation studies, they have all been implicated in neurodegenerative diseases. CAPZA2 is a subunit of an F-actin-capping complex and thus involved in the regulation of elongation of cytoskeletal filaments, playing a crucial role in a range of dynamic cellular processes [93]. Mutations in CAPZA2 can cause intellectual disability and developmental delay [94]. While the role of CAPZA2 in neurodegeneration is not well understood, it has been shown to be down-regulated in CSF of patients with Alzheimer’s disease [95]. Its transcript was shown to be up-regulated in the whole blood of ALS patients compared to controls [96]. ALDH16A1 belongs to the Aldehyde dehydrogenase superfamily. Aldehyde dehydrogenases constitute a key enzyme family involved in the detoxification of reactive aldehydes arising from endogenous metabolic processes and exogenous environmental sources, thereby mitigating cellular oxidative and electrophilic stress [97]. In Mast syndrome (SPG21), ALDH16A1 has been associated with masperdin, the protein in which causal mutations have been identified. Loss of ALDH16A1 function was hypothesized to be associated with a particular vulnerability of the upper motor neurons in this setting [98]. Data on the role of ALDH16A1 in ALS is, however, lacking. PFKL is a crucial enzyme in glycolysis. Glucose metabolism defects have been implicated in the pathogenesis of ALS: altered glucose metabolism has been shown in brain tissue of ALS patients as well as the SOD1 mouse model [99, 100]. PFKL is the liver-specific isoform of phosphofructokinase. While systemic glucose metabolism changes have been identified in patients with ALS [101], data on PFKL expression in ALS is lacking. The other isoforms (PFKM in muscle and PFKP in platelets) have been shown to be up-regulated at the transcript level in the spinal cord and induced pluripotent stem cell-derived motor neurons of ALS patients [102].

The initial findings and validation of our protein signature’s ability to classify ALS and control subjects are encouraging, though the diagnostic performance in a clinical setting is limited. Larger sample sizes and potentially additional proteins are likely necessary to develop more robust predictive models. We analyzed two independent cohorts from a single center, which limits the generalizability of our findings to other populations. Additionally, our discovery cohort was male-skewed, introducing a potential bias. To mitigate this, we modeled sex as covariate in the proteomics analysis. Importantly, the robustness of our findings is supported by the positive results observed in the independent validation cohort. In our study, we exclusively sampled tears with Schirmer test strips. While tears can also be collected using microcapillary tubes [103], both techniques are highly applicable for protein analyses [104]. A possible variability in protein composition should be considered [105, 106]. In TF, four proteins - LCN1, LYZ, LTF, and secretory immunoglobulin A - are known to account for 70–85% of total protein concentration [107, 108], thus potentially masking low abundant proteins that could have importance in ALS pathogenesis. This might explain why our proteomic analysis could not detect common biomarkers like neurofilaments in TF, although NfL has been described in the TF of Parkinson’s disease patients using the ultrasensitive Single Molecule Array (SIMOA) assay [28]. However, our data revealed an upregulation of cytoskeleton-associated proteins and pathways in the ALS group. The lack of significance of the other signature proteins in our Western blot validation approach might also be attributed to the biological variability within samples, compounded by technical limitations such as limited sample quantities. This might have reduced the robustness and sensitivity of the analyses. Studies in larger cohorts as well as using ultra-sensitive assays such as SIMOA or NUcleic acid Linked Immuno-Sandwich Assay (NULISA) may be needed to detect low abundant proteins reliably.

## Conclusions

In summary, TF is a well-accessible and non-invasively obtainable body fluid with substantial promise for biomarker discovery. In our study, we found prominent signals in proteins that have previously been implicated in the pathogenesis of ALS, particularly those related to neuroinflammation. We identified and experimentally validated six differentially abundant proteins - CRYM, PFKL, CAPZA2, ALDH16A1, SERPINC1, and HP. Notably, the combination of SERPINC1, HP, and CRYM demonstrated encouraging performance in disease classification. Future studies on larger and more diverse cohorts, including longitudinal sampling, will inform about the generalizability and value of these proteins as biomarkers for disease state and progression in a clinical setting.

## Supplementary Information

Below is the link to the electronic supplementary material.

Supplementary Material 1

Supplementary Material 2

Supplementary Material 3

Supplementary Material 4

Supplementary Material 5

## Data Availability

The source code for all computational analyses is available for download on GitHub (https://github.com/MendenLab/Tear-Fluid-Proteomics). Proteomic data were deposited to the ProteomeXchange Consortium via the PRIDE partner repository [109] with the dataset identifier PXD062971. Deidentified clinical data from this study are provided with a signed data access agreement upon request from the corresponding authors.
