# Supplementary material for: Identification and validation of a tear fluid-derived protein biomarker signature in patients with amyotrophic lateral sclerosis
Source: Acta Neuropathol Commun. 2025 Sep 2;13:187. doi: 10.1186/s40478-025-02109-6 (PMC12403336; doi:10.1186/s40478-025-02109-6)
Supplement: Supplementary file 1 — Supplementary Material 1 [file 40478_2025_2109_MOESM1_ESM.docx]

**Additional Information for**

**Identification and Validation of a Tear Fluid-Derived Protein Biomarker Signature in Patients with Amyotrophic Lateral Sclerosis**

Lena-Sophie Scholl^1,*^, Antonia F. Demleitner^2,*^, Jenny Riedel^3^, Seren Adachi^4^, Lisa Neuenroth^5^, Clara Meijs^6^, Laura Tzeplaeff^7^, Lucas Caldi Gomes^8^, Ana Galhoz^9^, Isabell Cordts^10^, Christof Lenz^11,†^, Michael Menden^12,†^*,* Paul Lingor^13,†^

1 Department of Neurology, TUM University Hospital Munich, Munich, Germany; mail: *ge68vel@mytum.de*

2 Department of Neurology, TUM University Hospital Munich, Munich, Germany; mail: *Antonia.demleitner@tum.de*
3 Computational Health Center, Helmholtz Munich, Neuherberg, Germany; Department of Biology, Ludwig-Maximilians University Munich, Martinsried, Germany; mail: *jy.riedel@web.de*

4 Department of Biochemistry and Pharmacology, Bio21 Molecular Science and Biotechnology Institute, The University of Melbourne, Parkville, Victoria, Australia; Melbourne Bioinformatics, Faculty of Medicine, Dentistry and Health Sciences, The University of Melbourne, Parkville, Victoria, Australia, mail: *adachis@student.unimelb.edu.au*

5 Department of Clinical Chemistry, University Medical Center Göttingen, Göttingen, Germany, mail: *lisa.neuenroth@med.uni-goettingen.de*

6 Computational Health Center, Helmholtz Munich, Neuherberg, Germany; Department of Biology, Ludwig-Maximilians University Munich, Martinsried, Germany; mail: *clara.meijs@i-med.ac.at*

7 Department of Neurology, TUM University Hospital Munich, Munich, Germany; mail: *laura.tzeplaeff@tum.de*

8 Department of Neurology, TUM University Hospital Munich, Munich, Germany; mail: *lucas.caldigomes@gmail.com*

9 Computational Health Center, Helmholtz Munich, Neuherberg, Germany; Department of Biology, Ludwig-Maximilians University Munich, Martinsried, Germany; mail: *ana.galhoz@helmholtz-munich.de*

10 Department of Neurology, TUM University Hospital Munich, Munich, Germany, mail: *isabell.cordts@tum.de*

11 Department of Clinical Chemistry, University Medical Center Göttingen, Göttingen, Germany, Bioanalytical Mass Spectrometry Group, Max Planck Institute for Multidisciplinary Sciences, Göttingen, Germany, mail: *christof.lenz@med.uni-goettingen.de*

12 Computational Health Center, Helmholtz Munich, Neuherberg, Germany, Department of Biochemistry and Pharmacology, Bio21 Molecular Science and Biotechnology Institute, The University of Melbourne, Parkville, Victoria, Australia, mail: *michael.menden@unimelb.edu.au*

13 Department of Neurology, TUM University Hospital Munich, Munich, Germany, German Center for Neurodegenerative Diseases (DZNE), Munich, Germany, Munich Cluster for Systems Neurology (SyNergy), Munich, Germany, mail: *paul.lingor@tum.de*

^†^ Corresponding Authors

*,^†^ These authors contributed equally to this work

**Additional Information - Table of Contents**

Supplementary Methods 3

Supplementary Figures 5

Supplementary Figure S1: Full-length tear fluid Western blots of the six targeted proteins. 5

Supplementary Figure S2: Western blots validating the identified tear fluid six-protein signature in all ALS and control samples. 7

Supplementary Figure S3: Heatmap of the normalized tear fluid protein expression for the discovery cohort in relation to demographic and clinical features. 9

Supplementary Figure S4: UMAPs of normalized tear fluid protein expression of all samples from the discovery cohort. 10

Supplementary Figure S5: UMAPs of normalized tear fluid protein expression for the ALS group only from the discovery cohort. 11

Supplementary Figure S6: STRING protein-protein interaction network analysis for significantly more abundant proteins. 12

Supplementary Figure S7: In-silico performance assessment of the six-protein signature based on Western blot validation (Ten runs). 13

Supplementary Figure S8: Assessment of quantitative reproducibility in DIA-MS data. 14

Supplementary Figure S9: Ranked TF protein abundance in controls and ALS subjects. 15

Supplementary Figure S10: AUROC line curve of nine proteins making up the best-performing model. 16

Supplementary Tables 17

Supplementary Table S1: Sensitivity and specificity of machine learning models to classify ALS based on the entire tear fluid proteome. 17

Supplementary Table S2: AUROC performance metrics of machine learning models using HP, SERPINC1, and a combination of both for the prediction of ALS versus controls. 17

Supplementary Data 18

Supplementary Data S1: Detailed results of differential abundance analysis conducted with PERSEUS. 18

Supplementary Data S2: Detailed gene set enrichment analysis results with protein assignment to specific pathways. 18

Supplementary Data S3: Tier list of proteins that were considered as putative biomarker proteins for further validation. 18

Supplementary Data S4: Detailed ranked protein abundance. 19

# Supplementary Methods

**Identification of the tear fluid-derived protein signature leveraging machine learning in detail**

To evaluate the capability of TF proteins to distinguish ALS patients from controls, we leveraged supervised machine learning algorithms implemented via the caret package (v6.0-94^1^) in R (v4.2.3^2^). Four machine learning algorithms were applied, including linear regression (lasso), random forest, and SVM with radial and linear kernels, to predict ALS status based on the entire TF proteome of ALS and control subjects. A nested 10-fold cross-validation with 500 iterations of bootstrapping was employed to strengthen robustness.

Proteins weighted by the linear regression model, indicating a high importance in ALS prediction during the bootstrapping procedure, were extracted and compiled to a tier list (**Data S3)**. These putative biomarker proteins were ranked upon specific criteria, focusing on a low bootstrapping FDR and high average weight from machine learning analysis, as well as a high -log10 FDR and absolute log2 FC values from the differential abundance analysis. The bootstrapping FDR of each protein was calculated with the RobustRankAggreg package^3^ and was based on the selection frequency and rank of the absolute weights from the linear regression model.

Next, lasso regression models based only on the differentially abundant proteins from the aforementioned tier list were created using the previously estimated protein weights. Based on the results of the tier list and for the sake of translating the TF-derived protein signature into clinical routine, we manually restricted the number of protein combinations to nine: HP, CRYM, ALDH16A1, mannose-1-phosphate guanyltransferase alpha (GMPPA), PFKL, eukaryotic translation initiation factor 2 subunit 2 (EIF2S2), SERPINC1, secretoglobin family 1D member 1 (SCGB1D1) and CAPZA2. Possible combinations of these nine putative biomarker proteins were tested and their discriminatory performance was assessed by AUROC to identify a signature that most effectively classifies ALS and controls.

The best-performing protein signature consisted of nine proteins: HP, CRYM, ALDH16A1, mannose-1-phosphate guanyltransferase alpha (GMPPA), PFKL, eukaryotic translation initiation factor 2 subunit 2 (EIF2S2), SERPINC1, and secretoglobin family 1D member 1 (SCGB1D1) and CAPZA2 (**Figure S10**). Due to the lack of availability of reproducibly working antibodies and protein detection limitations, the combination of six proteins (CRYM, CAPZA2, ALDH16A1, PFKL, SERPINC1, and HP) was selected and validated by Western blot in TF (**Figure 3 b-g, Figure S2**).

##### **Ranked protein abundance**

Rstudio (version 2024.12.0.467^4^; R version 4.4.2 (2024-10-31), R Core Team^2^) was used to analyze and visualize the ranked protein abundance. Log2-transformed intensity values derived from PERSEUS analysis were extracted for two technical replicates of each ALS and control subject for the 876 proteins consistently abundant in at least 66% of the samples. Log2-transformation was reversed, and the mean intensity of the two technical replicates was calculated. Subsequently, the mean for all controls and ALS patients was determined separately for each protein and log10-transformed. All 876 proteins were ranked in ascending order based on the mean abundance in ALS or controls, with the highest rank assigned to the most abundant protein (**Figure S9,** **Data S4**).

### **Supplementary References**

1. Kuhn M. Building Predictive Models in R Using the caret Package. Journal of Statistical Software. 2008;Vol. 28(Issue 5).

2. Team RC. R: A Language and Environment for Statistical Computing Vienna, Austria: R Foundation for Statistical Computing; 2023 [Available from: https://www.R-project.org/.

3. Kolde R, Laur, S. RobustRankAggreg: Methods for Robust Rank Aggregation, R package version 1.1. 2022 [Available from: https://cran.r-project.org/web/packages/RobustRankAggreg/index.html.

4. Team R. RStudio: Integrated Development for R Boston: RStudio; 2023 [Available from: http://www.rstudio.com/.

# Supplementary Figures

##### **
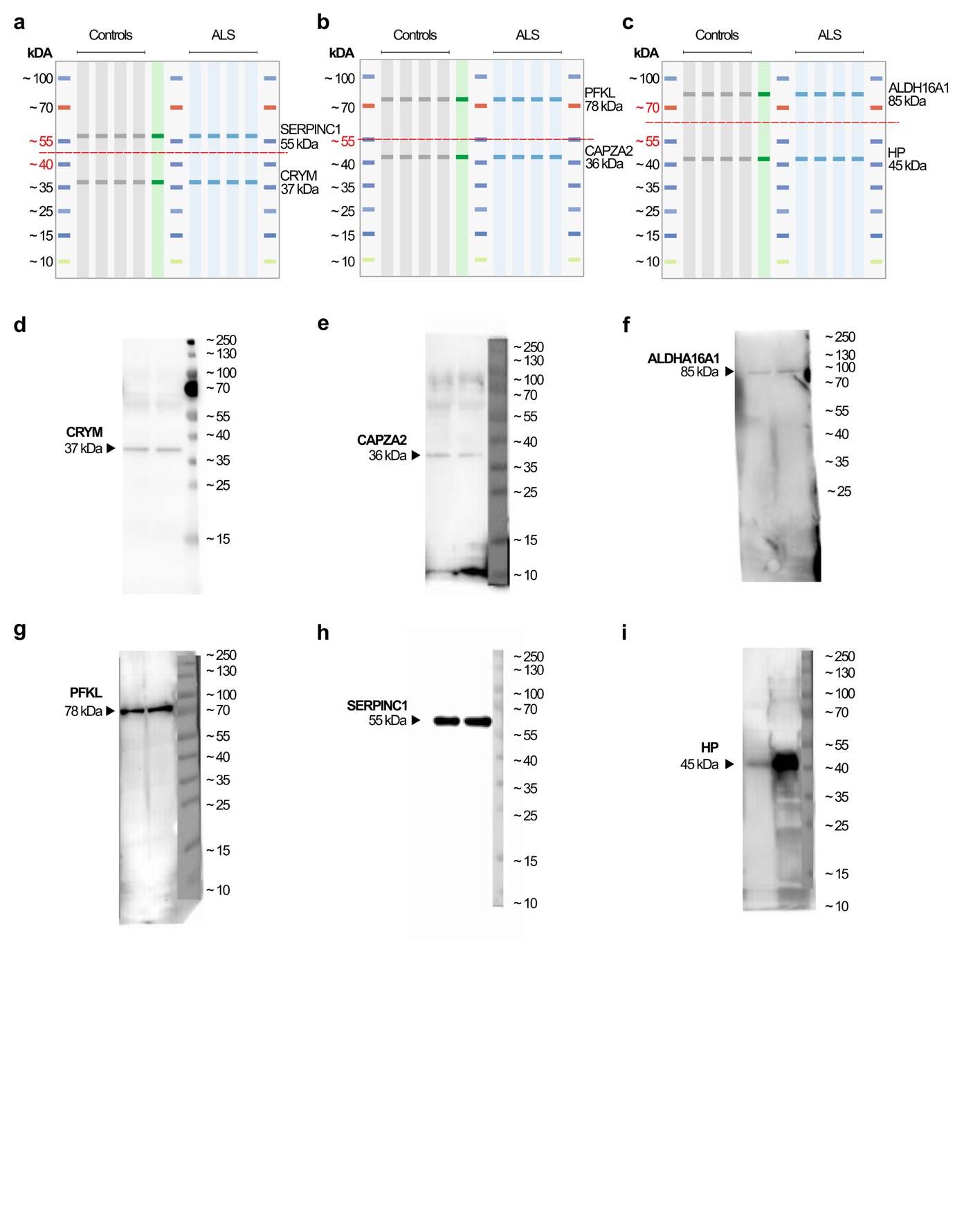
**

## Supplementary Figure S1: Full-length tear fluid Western blots of the six targeted proteins.

**a-c** Experimental setup for membrane sectioning for the detection of the six proteins. Each ALS (blue) and control (grey) sample was loaded in triplicates for gel electrophoresis, along with a pooled standard (green) for protein normalization. Following protein transfer, membranes were sectioned at specific molecular weight levels, as indicated by the prestained protein ladder. The red line marks the sectioning level. Membrane one was approximately cut at 50 kDa to detect CRYM (~ 37 kDa) and SERPINC1 (~ 55 kDa). Membrane two was sectioned at 55 kDa for CAPZA2 (~36 kDa) and PFKL (~78 kDa). Membrane three at approximately 60 kDa to analyze HP (~45 kDa) and ALDH16A1 (~85 kDa).

**d-i** Full-length Western blot of CRYM, CAPZA2, ALDH16A1, PFKL, SERPINC1, and HP. A protein ladder (pre-stained MW marker) is shown on the right. Specific bands were detected at an expected MW of approximately 37 kDa for CRYM, 36 kDa for CAPZA2, 85 kDa for ALDH16A1, 78 kDa for PFKL, 55 kDa for SERPINC1, and 45 kDa for HP.

**Abbreviations:** ALS, Amyotrophic lateral sclerosis; CRYM, mu-crystallin; CAPZA2, f-actin-capping protein subunit alpha-2; ALDH16A1, aldehyde dehydrogenase family 16 member A1; PFKL, phosphofructokinase, liver type; SERPINC1, antithrombin-III; HP, haptoglobin, MW, molecular weight.

##### **
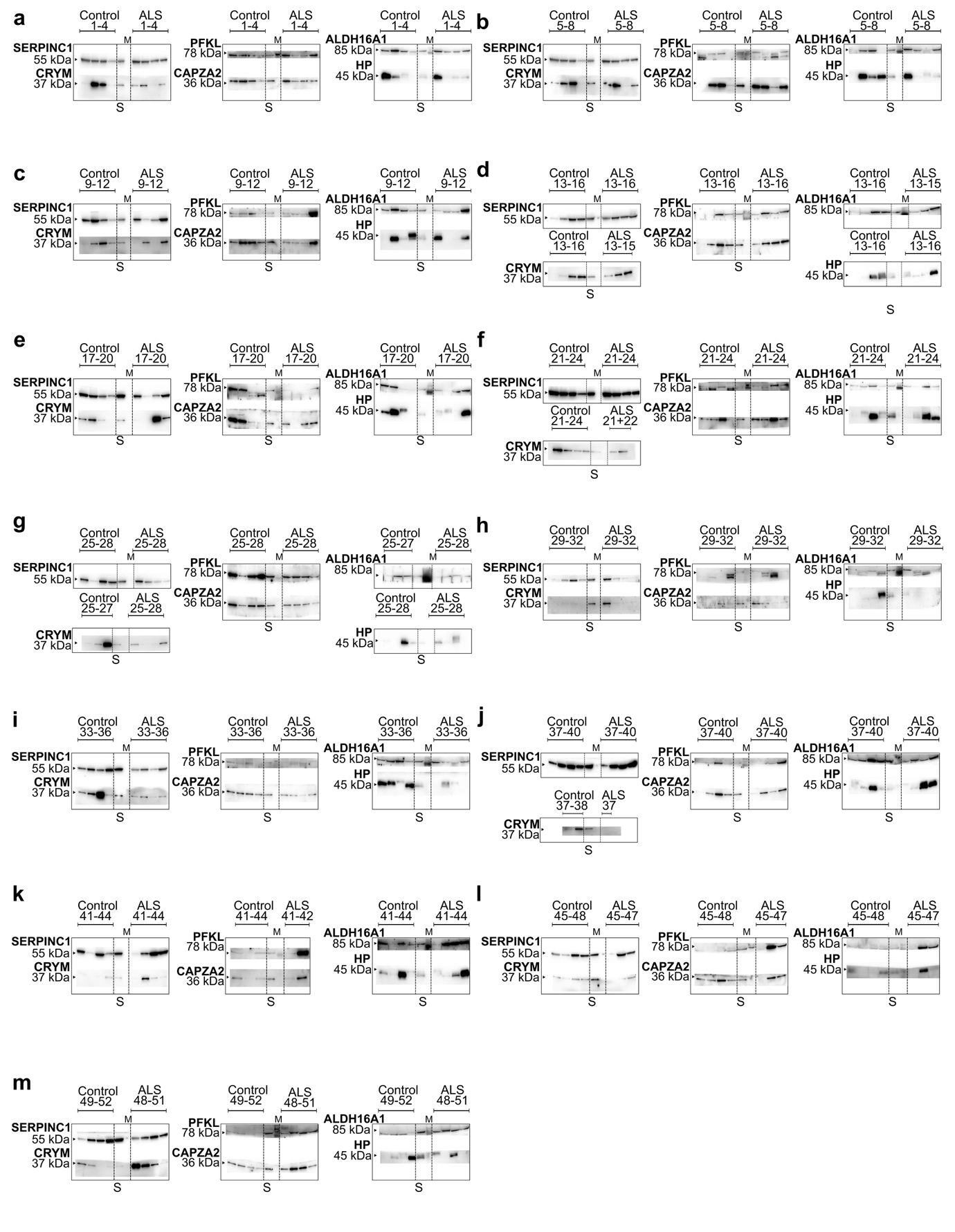
**

## Supplementary Figure S2: Western blots validating the identified tear fluid six-protein signature in all ALS and control samples.

**a-m** Compilation of all Western blot membranes for the validation of the six-protein signature in ALS patients (*n* = 51) and controls (*n* = 51). Each sample was loaded in triplicates for the detection of the six proteins. A pooled TF standard (S) served as normalization. For protein detection, membranes were sectioned at specific molecular weights as indicated by the marker (M) (**Figure S1 a-i**).

**Abbreviations:** TF, tear fluid; ALS, Amyotrophic lateral sclerosis; S, standard; M, marker; CRYM, mu-crystallin; CAPZA2, f-actin-capping protein subunit alpha-2; ALDH16A1, aldehyde dehydrogenase family 16 member A1; PFKL, phosphofructokinase, liver type; SERPINC1, antithrombin-III; HP, haptoglobin.

##### **
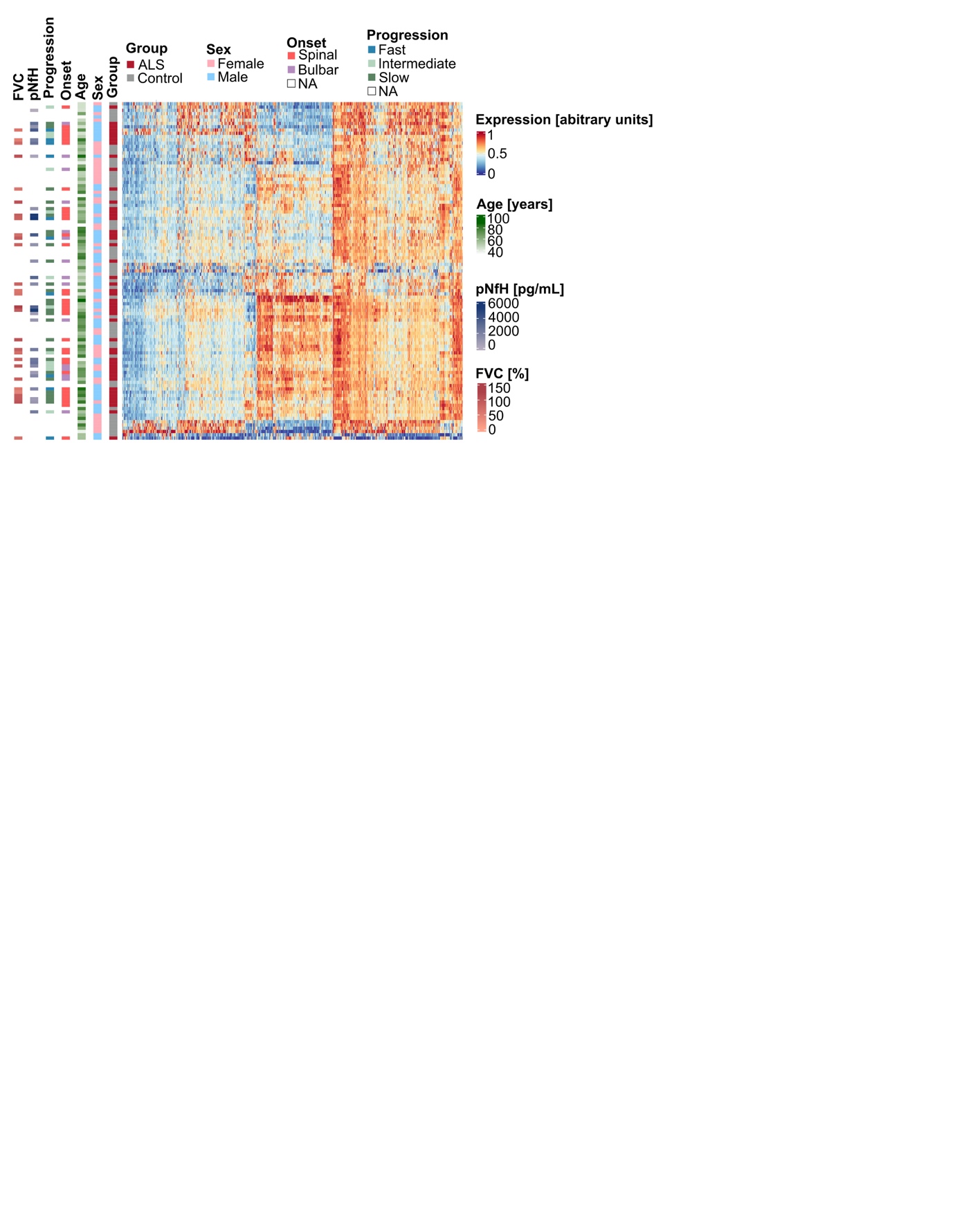
**

## Supplementary Figure S3: Heatmap of the normalized tear fluid protein expression for the discovery cohort in relation to demographic and clinical features.

Heatmap of normalized TF protein abundance in the discovery cohort, with unsupervised hierarchical clustering of 876 proteins. Rows represent proteins, and columns represent samples. Clinical features, including sex, age [years], onset, progression rate, CSF pNfH levels [pg/mL], and FVC [%], are annotated. Data are sorted for similar protein abundance patterns.
**Abbreviations:** TF, tear fluid; ALS, Amyotrophic lateral sclerosis; CSF, cerebrospinal fluid; pNfH, phosphorylated neurofilament heavy chain; FVC, forced vital capacity; NA, not applicable.

##### **
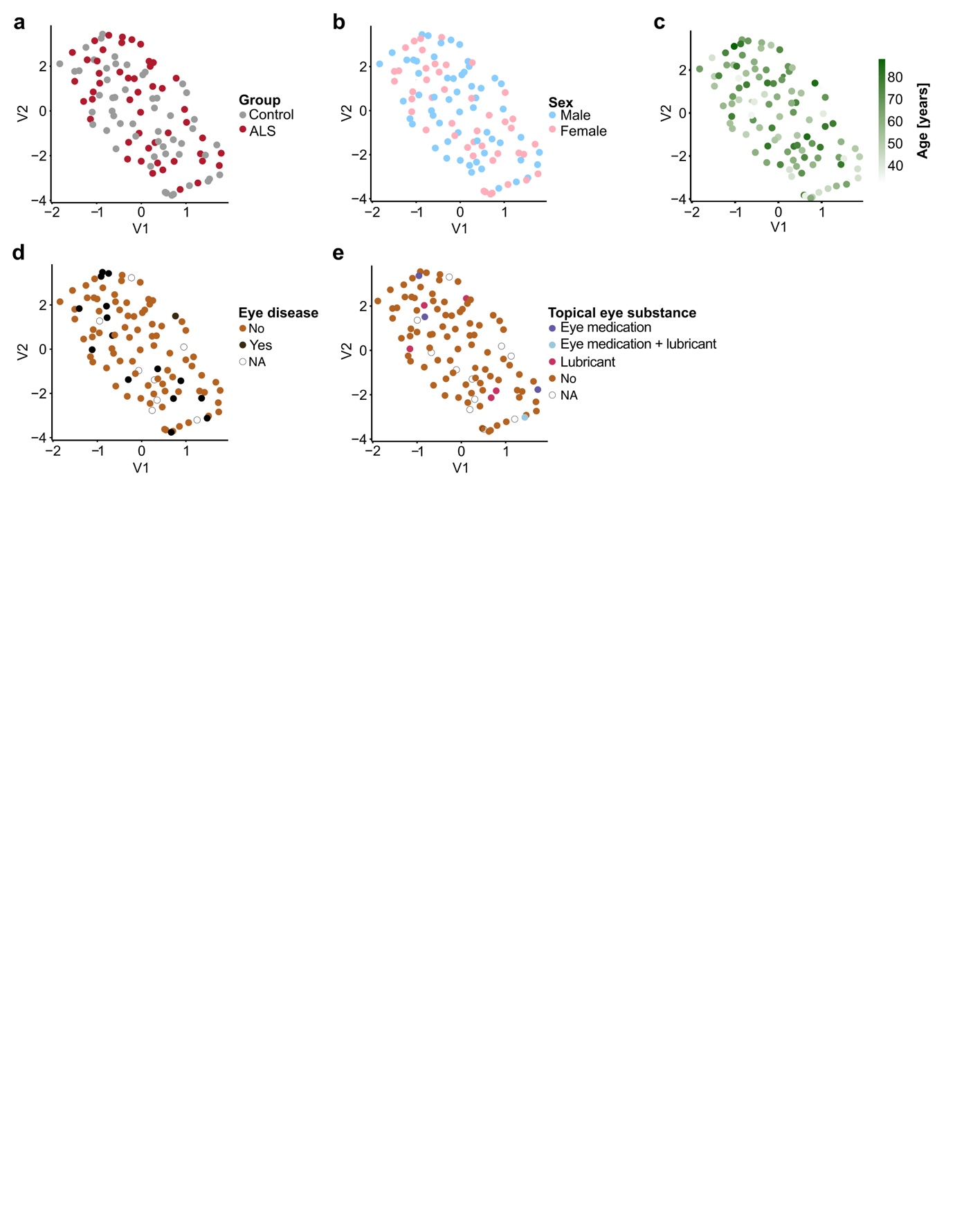
**

## Supplementary Figure S4: UMAPs of normalized tear fluid protein expression of all samples from the discovery cohort.

**a-e** Circle colors represent the group (ALS and controls), sex, age [years], presence of eye disease, and use of topical eye substances. Principal components 1 (V1) and 2 (V2) are mapped to x- and y-axis, respectively.

**Abbreviations:** UMAP, uniform manifold approximation and projection; TF, tear fluid; ALS, Amyotrophic lateral sclerosis; NA, not applicable.

##### **
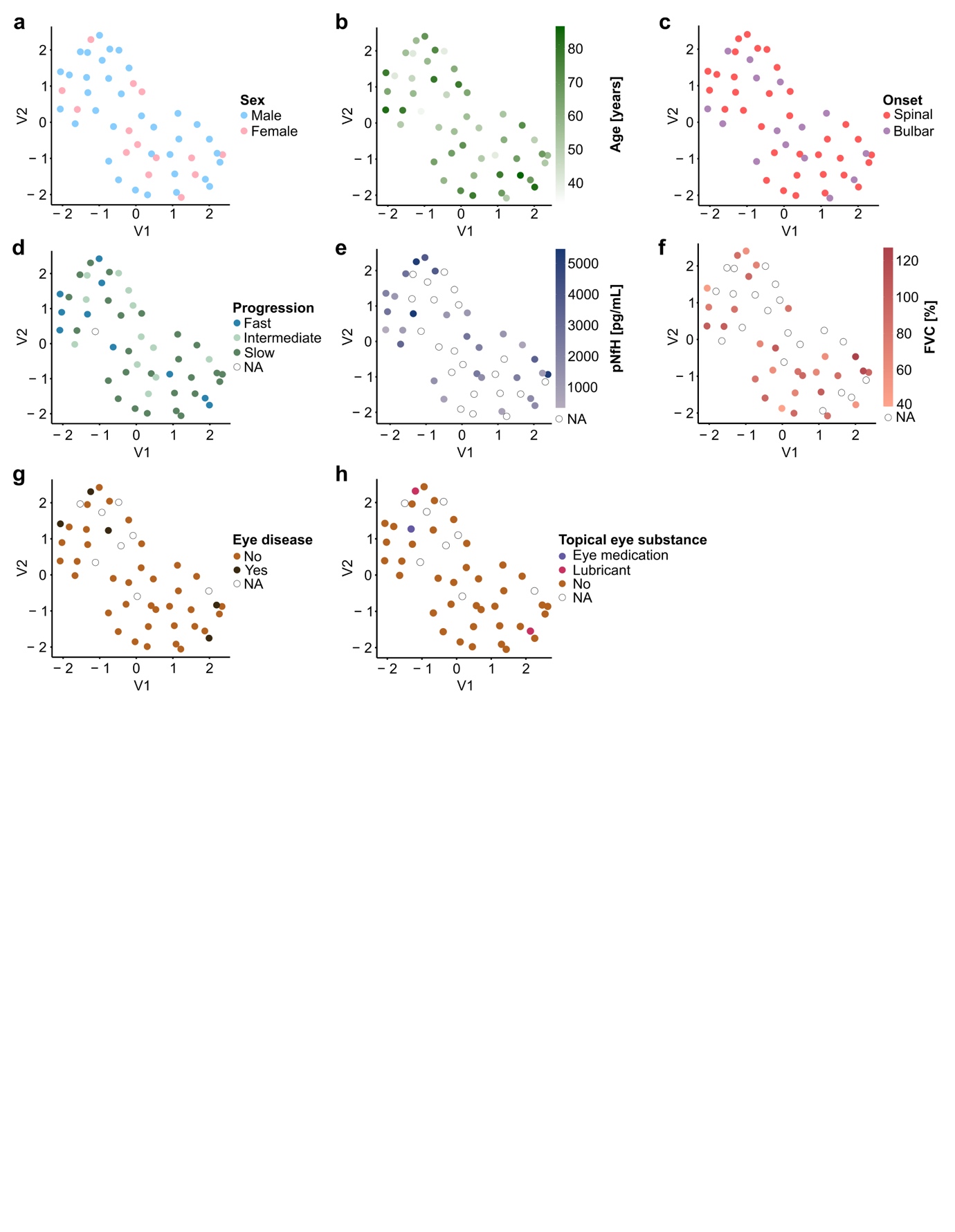
**

## Supplementary Figure S5: UMAPs of normalized tear fluid protein expression for the ALS group only from the discovery cohort.

**a-h** Circle colors represent the sex, age [years], stratum of onset, progression rate, CSF pNfH level [pg/mL], FVC [%], presence of eye disease, and the use of topical eye substances. Principal components 1 (V1) and 2 (V2) are mapped to x- and y-axis, respectively.

**Abbreviations:** UMAP, uniform manifold approximation and projection; TF, tear fluid; ALS, Amyotrophic lateral sclerosis; CSF, cerebrospinal fluid; pNfH, phosphorylated neurofilament heavy chain; FVC, forced vital capacity; NA, not applicable.

##### **
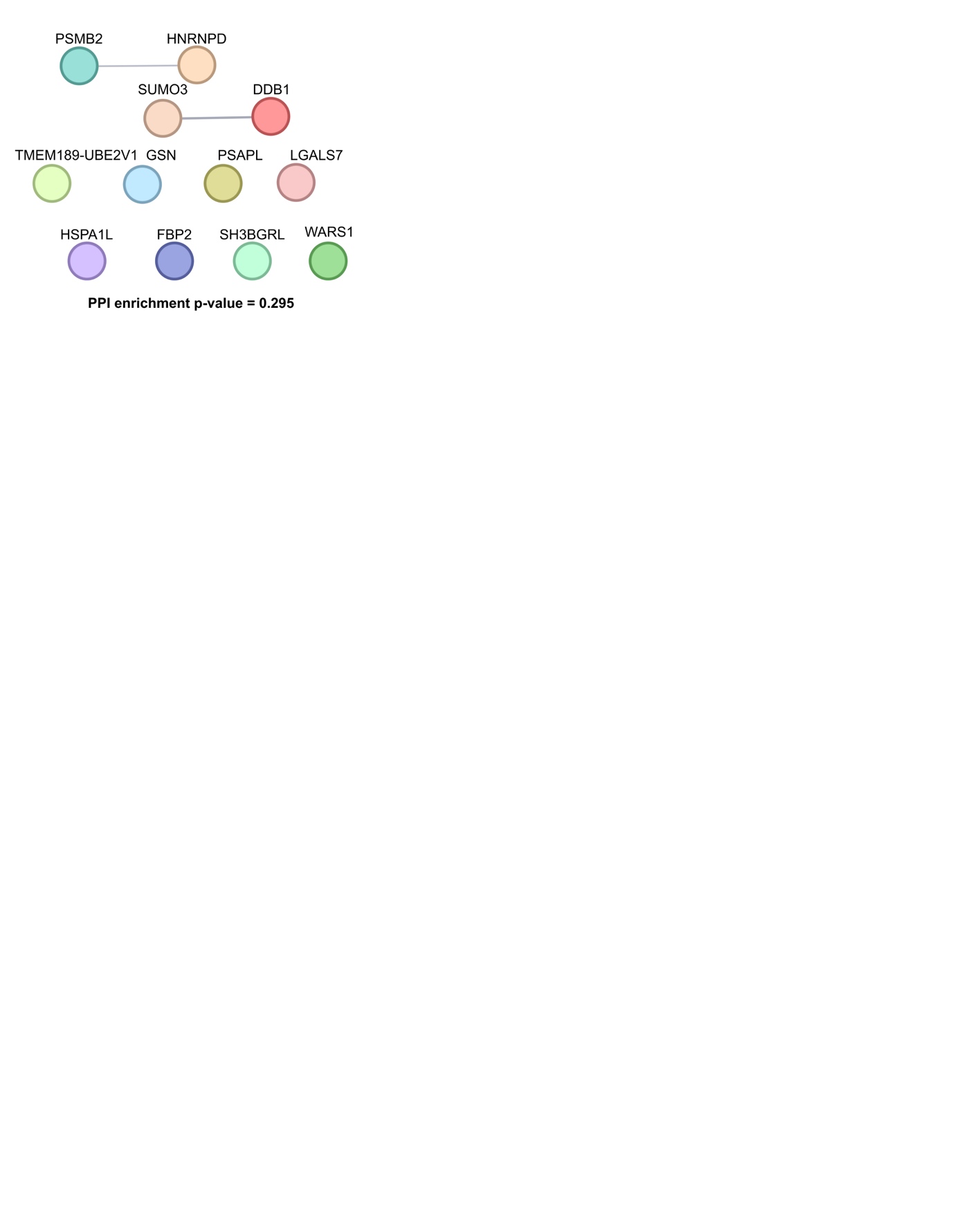
**

## Supplementary Figure S6: STRING protein-protein interaction network analysis for significantly more abundant proteins.

##### Node color is randomly assigned by default of the STRING platform v12.0. The PPI enrichment p-value = 0.295 for the entire network is stated below.

**Abbreviation:** PPI, protein-protein interaction.

##### **
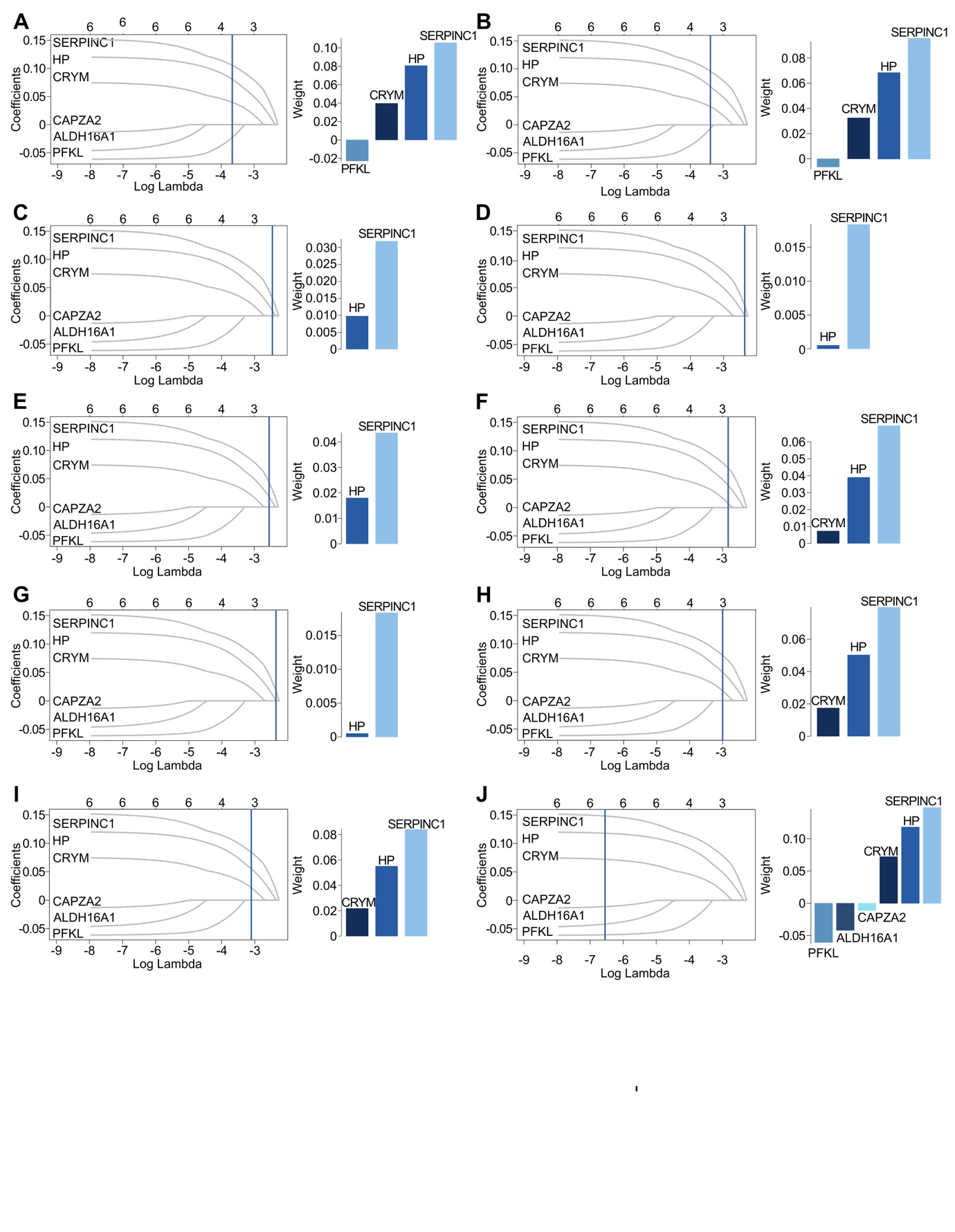
**

## Supplementary Figure S7: In-silico performance assessment of the six-protein signature based on Western blot validation (Ten runs).

**a-j** Parameterization of L1 regularization for lasso in nine models from the 10-fold cross-validation (left) and bar chart of the selected proteins in the corresponding lasso model with high impact in the ALS classification (right). The x-axis represents the log-transformed lambda values, while the y-axis indicates the model coefficients. The best-performing lambda is indicated (blue line). The bar chart shows the weights (y-axis) assigned to each protein (x-axis) in the chosen lasso model.

**Abbreviations:** CRYM, mu-crystallin; CAPZA2, f-actin-capping protein subunit alpha-2; ALDH16A1, aldehyde dehydrogenase family 16 member A1; PFKL, phosphofructokinase, liver type; SERPINC1, antithrombin-III; HP, haptoglobin.

## Supplementary Figure S8: Assessment of quantitative reproducibility in DIA-MS data.

**a** Protein identification completeness across all samples. The x-axis represents individual samples (including technical and biological replicates), and the y-axis indicates the total number of proteins identified per sample. Nearly 1,000 proteins out of 2,205 were detected in at least 60% of the samples, reflecting strong overall data completeness.

**b** Protein-level coefficient of variation (CV) across all samples. The x-axis represents individual proteins, and the y-axis shows the mean CV (%) for each protein. The majority of the proteins exhibited a CV below 50% (1,588 out of 2,205), indicating a good reproducibility of quantification. Proteins highlighted in orange (CRYM, PFKL, CAPZA2, ALDH16A1, SERPINC1, and HP) represent the proposed biomarker signature. All six proteins fall within a CV range of 25–50%, supporting their quantitative robustness and potential utility as biomarkers.

**Abbreviations:** CRYM, mu-crystallin; CAPZA2, f-actin-capping protein subunit alpha-2; ALDH16A1, aldehyde dehydrogenase family 16 member A1; PFKL, phosphofructokinase, liver type; SERPINC1, antithrombin-III; HP, haptoglobin.

##### **
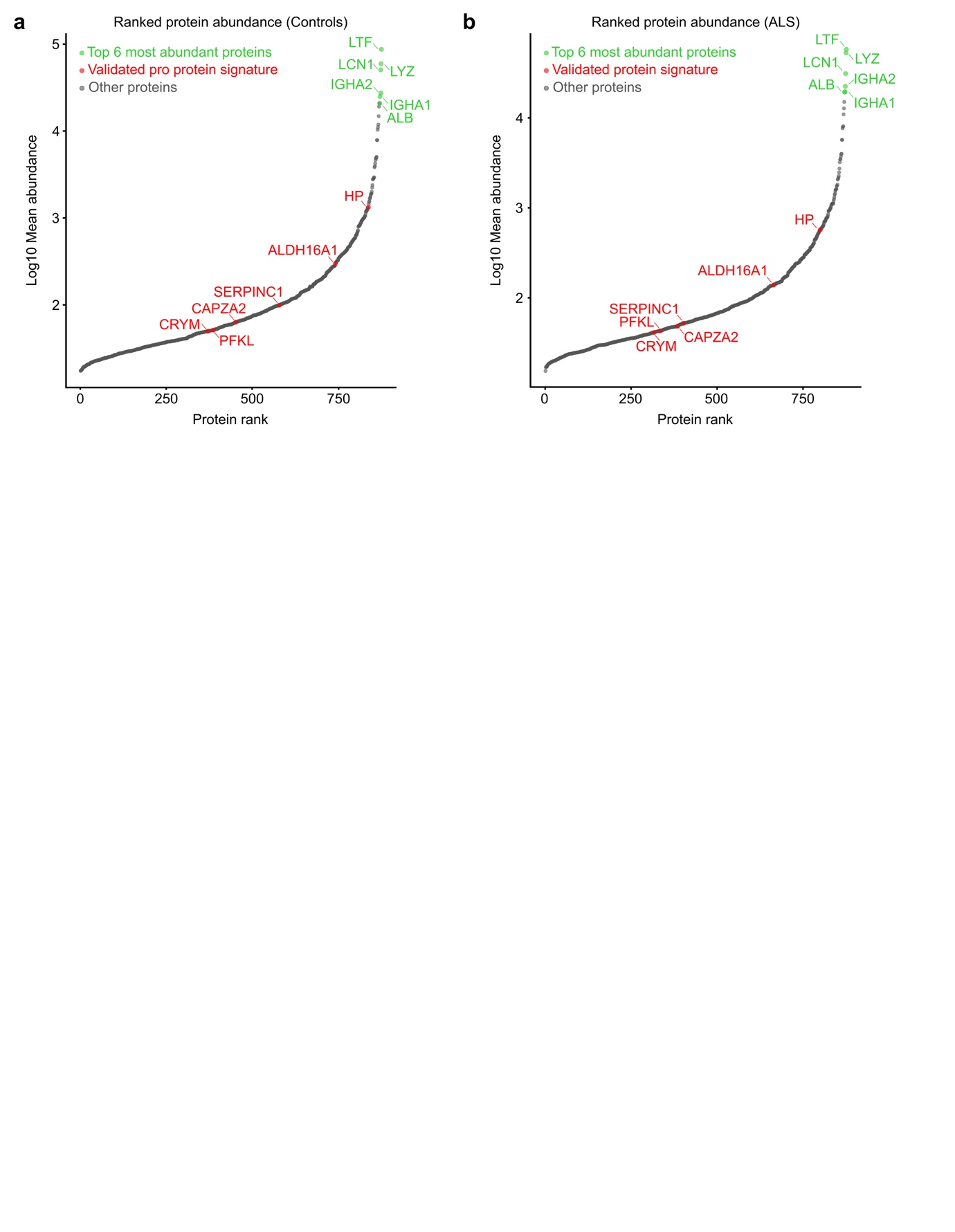
**

## Supplementary Figure S9: Ranked TF protein abundance in controls and ALS subjects.

**a-b** Ranked protein abundance plot of proteins ordered by their log₁₀ mean abundance in the TF of control (left) and ALS (right) subjects. The x-axis shows the protein rank, and the y-axis depicts the corresponding log10 mean abundance. Proteins were ranked increasingly (**Data S4**).

**Abbreviations:** ALS, Amyotrophic lateral sclerosis; CRYM, mu-crystallin, PFKL, phosphofructokinase, liver type; CAPZA2, f-actin-capping protein subunit alpha-2; SERPINC1, antithrombin-III; ALDH16A1, aldehyde dehydrogenase family 16 member A1; HP, haptoglobin; ALB; serum albumin; IGHA1, immunoglobulin heavy constant alpha 1; IGHA2, immunoglobulin heavy constant alpha 2; LCN1, lipocalin-1; LYZ, lysozyme C; LTF, lactotransferrin.

##### **
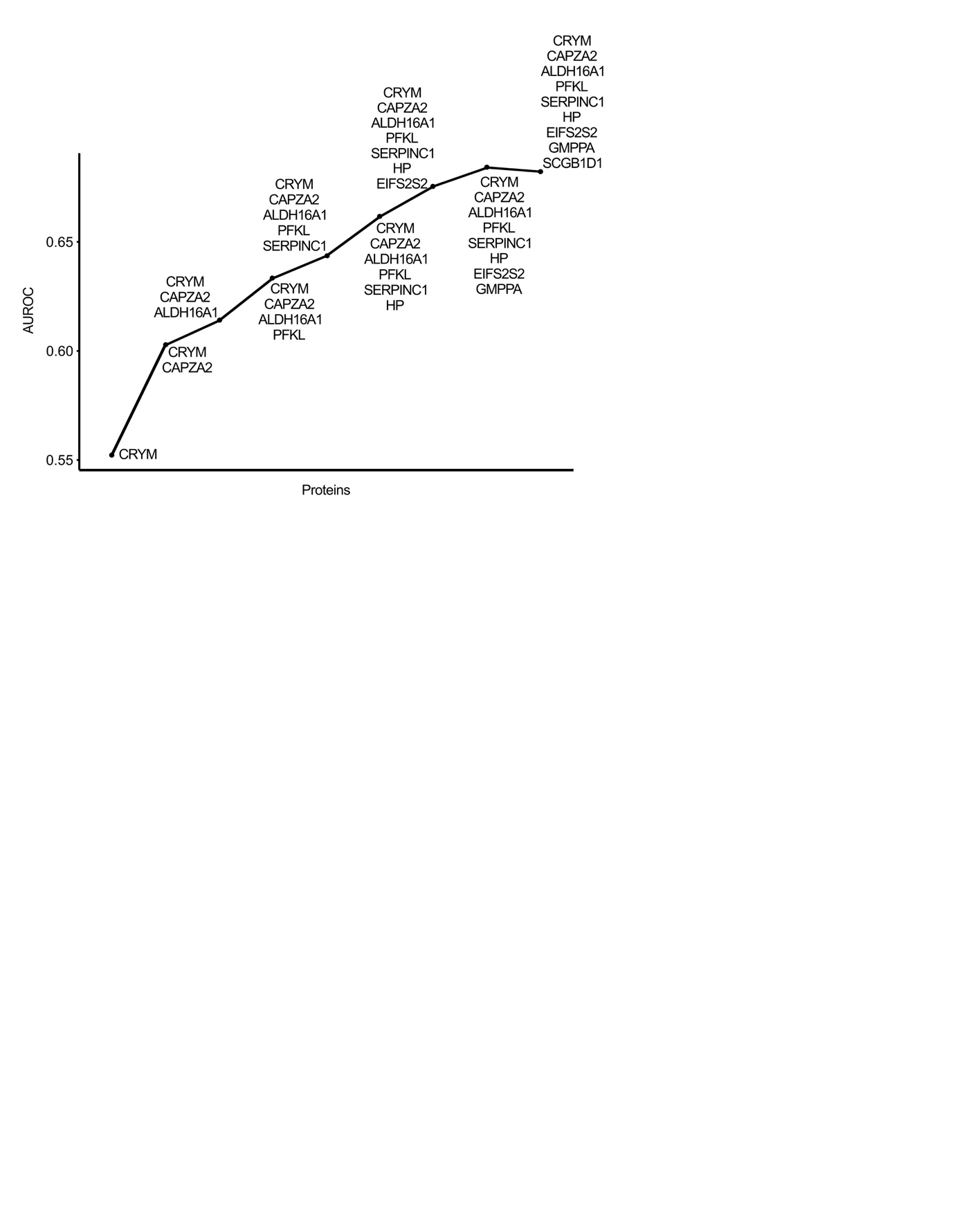
**

## Supplementary Figure S10: AUROC line curve of nine proteins making up the best-performing model.

The x-axis displays the proteins considered as putative biomarker proteins to differentiate ALS from controls, while the y-axis represents the corresponding AUROC values, indicating their discriminatory performance.

**Abbreviations:** AUROC, area under the receiver operating characteristics; CRYM, mu-crystallin; CAPZA2, f-actin-capping protein subunit alpha-2; ALDH16A1, aldehyde dehydrogenase family 16 member A1; PFKL, phosphofructokinase, liver type; SERPINC1, antithrombin-III; HP, haptoglobin; EIF2S2, eukaryotic translation initiation factor 2 subunit 2; GMPPA, mannose-1-phosphate guanyltransferase alpha; SCGB1D1, secretoglobin family 1D member 1.

# Supplementary Tables

| **Machine learning algorithm** | **Sensitivity [%]** | **Error**  **sensitivity [%]** | **Specificity [%]** | **Error**  **specificity [%]** |
| --- | --- | --- | --- | --- |
| SVM linear kernel | 80.79 | 11.11 | 73.59 | 9.63 |
| SVM radial kernel | 78.46 | 11.02 | 71.44 | 10.25 |
| Random forest | 73.81 | 12.02 | 59.30 | 10.89 |
| Linear regression (lasso) | 72.68 | 11.92 | 56.54 | 10.45 |

## Supplementary Table S1: Sensitivity and specificity of machine learning models to classify ALS based on the entire tear fluid proteome.

**Abbreviations:** SVM, support vector machine.

|  | **AUROC [%] of machine learning algorithms** | | | |
| --- | --- | --- | --- | --- |
| **Proteins** | **SVM with linear kernel** | **SVM with radial kernel** | **Linear regression** | **Random forest** |
| SERPINC1 | 57.18 | 49.63 | 56.32 | 54.34 |
| HP | 57.45 | 49.97 | 56.55 | 54.53 |
| HP, SERPINC1 | 59.90 | 52.82 | 57.50 | 58.91 |

## Supplementary Table S2: AUROC performance metrics of machine learning models using HP, SERPINC1, and a combination of both for the prediction of ALS versus controls.

**Abbreviations:** AUROC, area under the receiver operating characteristic curve; SVM, support vector machine, SERPINC1, antithrombin-III; HP, haptoglobin.

# Supplementary Data

## Supplementary Data S1: Detailed results of differential abundance analysis conducted with PERSEUS.

Summary of the differential abundance analysis results (DAA) for all identified tear fluid proteins, comparing their abundance levels between ALS and control samples. The analysis was performed using PERSEUS (v.1.6.15.0). Significant threshold was determined applying the standard settings, including with a false discovery rate (FDR) < 0.1. Proteins with significantly higher abundance in ALS are colored orange, while those with significantly lower abundance are blue. Proteins with no significant differential abundance between ALS and control samples are uncolored (white). Proteins identified as significantly differentially abundant are marked with a '+' in the first column, whereas those with no significant differential abundance are labeled with a '-'. Each protein is annotated with its -log10 FDR and log2 fold change (log2FC) derived from the DAA. Protein abbreviations and descriptions were obtained from the UniProtKB Homo sapiens reference proteome following mass spectrometry analysis.

**Abbreviations:** FDR, false discovery rate; FC, fold change.

## Supplementary Data S2: Detailed gene set enrichment analysis results with protein assignment to specific pathways.

Overview of all identified tear fluid (TF) proteins in ALS and control samples, mapped to Gene Ontology (GO) pathways and corresponding GO categories: biological process (BP), cellular component (CC), and molecular function (MF). The annotation of specific pathways to broader generic categories is provided, along with key metrics, including the enrichment score (ES), normalized enrichment score (NES), and statistical measures such as p-value, adjusted p-value, and q-value. Additionally, the set size of each GO pathway and the number of identified proteins assigned to these pathways are reported.

**Abbreviations:** GO, gene ontology; BP, biological process; CC, cellular component; MF, molecular function; ES, enrichment score; NES, normalized enrichment score; N, number.

## Supplementary Data S3: Tier list of proteins that were considered as putative biomarker proteins for further validation.

A compilation of proteins weighted by linear regression algorithm to differentiate between ALS and control samples based on the entire TF proteome was generated. These proteins were ranked upon machine learning analysis results, specifically considering a low bootstrapping (BS) FDR, high average weight in the BS linear regression model, and low BS error. Additionally, the ranking incorporated results from differential abundance analysis (DAA), prioritizing proteins with a high -log10 ​false discovery rate (FDR) and a high absolute log2fold change (FC). Proteins exhibiting significantly differential abundance in ALS compared to controls are labeled with ‘+’, while those showing no significant differential abundance are labeled with ‘-’.

**Abbreviations:** avg, average; LR, linear regression; BS, bootstrapping; FDR, false discovery rate; FC, fold change; DAA, differential abundance analysis.

## Supplementary Data S4: Detailed ranked protein abundance.

Ranked protein abundance was performed by considering the log10-transformed mean abundance of all ALS and control subjects separately for the 876 proteins that were abundant in at least 66% of all samples. Log10 Mean abundance per protein was sorted ascendingly so that the highest rank (876) corresponds to the most abundant protein. The six most abundant proteins (green) and the six proteins of our validated protein signature (red) are labeled.

**Abbreviations:** CTRL, control; ALS; Amyotrophic lateral sclerosis.
